# The RIO trial: rationale, design, and the role of community involvement in a randomised placebo-controlled trial of antiretroviral therapy plus dual long-acting HIV-specific broadly neutralising antibodies (bNAbs) in participants diagnosed with recent HIV infection—study protocol for a two-stage randomised phase II trial

**DOI:** 10.1186/s13063-022-06151-w

**Published:** 2022-04-05

**Authors:** Ming Jie Lee, Simon Collins, Daphne Babalis, Nicholas Johnson, Emanuela Falaschetti, A. Toby Prevost, Ambreen Ashraf, Milaana Jacob, Tom Cole, Lisa Hurley, Matthew Pace, Ane Ogbe, Maryam Khan, Panagiota Zacharopoulou, Helen Brown, Euan Sutherland, Hanna Box, Julie Fox, Steven Deeks, Jill Horowitz, Michel C. Nussenzweig, Marina Caskey, John Frater, Sarah Fidler

**Affiliations:** 1 Department of Infectious Disease, Imperial College London, London, UK; 2 HIV i-Base, London, UK; 3 Imperial Clinical Trials Unit, School of Public health, Imperial College London, London, UK; 4 King’s Clinical Trials Unit, King’s College London, London, UK; 5 NIHR Imperial Clinical Research Facility, Imperial College London, London, UK; 6 Peter Medawar Building for Pathogen Research, University of Oxford, Oxford, UK; 7 Imperial College Clinical Trials Centre, Imperial College Healthcare NHS Trust, London, UK; 8 Harrison Wing, Guy’s and St Thomas Hospital NHS Foundation Trust, London, UK; 9 Department of Medicine, University of California, San Francisco, CA 94110 USA; 10 Laboratory of Molecular Immunology, The Rockefeller University, New York, USA

**Keywords:** HIV, Primary infection, Broadly neutralising antibodies, Antiretroviral therapy, Virological remission, T cell Immunity

## Abstract

**Background:**

Antiretroviral therapy (ART) has led to dramatic improvements in survival for people living with HIV, but is unable to cure infection, or induce viral control off therapy. Designing intervention trials with novel agents with the potential to confer a period of HIV remission without ART remains a key scientific and community goal. We detail the rationale, design, and outcomes of a randomised, placebo-controlled trial of two HIV-specific long-acting broadly neutralising antibodies (bNAbs): 3BNC117-LS and 10-1074-LS, which target CD4 binding site and V3 loop respectively, on post-treatment viral control.

**Methods:**

RIO is a randomised, placebo-controlled, double-blinded prospective phase II study. Eligible individuals will have started ART within 3 months of primary HIV infection and have viral sequences that appear to be sensitive to both bNAbs. It will randomise 72 eligible participants 1:1 to the following arms via a two-stage design. In Stage 1, arm A participants are given dual long-acting (LS-variants) bNAbs infusions, followed by intensively monitored Analytical Treatment Interruption (ATI) (*n* = 36); in arm B, participants receive placebo infusions followed by ATI. The primary endpoint will be time to viral rebound within 36 weeks after ATI. Upon viral rebound, the participant and researcher are unblinded. Participants in arm A recommence ART and complete the study. Participants in arm B are invited to restart ART and enroll into Stage 2 where they will receive open-label LS bNAbs, followed by a second ATI 24 weeks after. Secondary and exploratory endpoints include adverse events, time to undetectable viraemia after restarting ART, immunological markers, HIV proviral DNA, serum bNAb concentrations in blood, bNAb resistance at viral rebound, and quality of life measures.

**Discussion:**

The two-stage design was determined in collaboration with community involvement. This design allows all participants the option to receive bNAbs. It also tests the hypothesis that bNAbs may drive sustained HIV control beyond the duration of detectable bNAb concentrations. Community representatives were involved at all stages. This included the two-stage design, discussion on the criteria to restart ART, frequency of monitoring visits off ART, and reducing the risk of onward transmission to HIV-negative partners. It also included responding to the challenges of COVID-19.

**Trial registration:**

The protocol is registered on Clinical.trials.gov and EudraCT and has approval from UK Ethics and MHRA.

**Supplementary Information:**

The online version contains supplementary material available at 10.1186/s13063-022-06151-w.

## Administrative information

Note: the numbers in curly brackets in this protocol refer to SPIRIT checklist item numbers. The order of the items has been modified to group similar items (see http://www.equator-network.org/reporting-guidelines/spirit-2013-statement-defining-standard-protocol-items-for-clinical-trials/).

**Table:** 

Title {1}	The RIO trial: Rationale, design, and the role of community involvement in a randomised placebo-controlled trial of antiretroviral therapy plus dual long-acting HIV-specific broadly neutralising antibodies (bNAbs) in participants diagnosed with recent HIV infection - Study protocol for a two-stage randomised trial
Trial registration {2a and 2b}.	EUDRACT Number: 2019-002129-31 Registered 26^th^ September 2019 ISRCTN Number / Clinical trials.gov Number: NCT04319367 https://clinicaltrials.gov/ct2/show/NCT04319367?term=NCT04319367&draw=2&rank=1 Registered 24^th^ March 2020
Protocol version {3}	Protocol Version 5.0 Date 19/10/2021
Funding {4}	This research is funded by the Bill and Melinda Gates Foundation, P.O. Box 23350, Seattle, WA 9810, USA.
Author details {5a}	Ming Jie Lee: Department of Infectious Disease, Imperial College London, UK Simon Collins: HIV i-Base, UK Daphne Babalis: Imperial Clinical Trials Unit, School of Public health, Imperial College London, UK Nicholas Johnson: Imperial Clinical Trials Unit, School of Public health, Imperial College London, UK Emanuela Falaschetti: Imperial Clinical Trials Unit, School of Public health, Imperial College London, UK A Toby Prevost: King’s Clinical Trials Unit, King’s College London, UK Ambreen Ashraf: Imperial Clinical Trials Unit, School of Public health, Imperial College London, UK Milaana Jacob: Imperial Clinical Trials Unit, School of Public health, Imperial College London, UK Tom Cole: NIHR Imperial Clinical Research Facility, Imperial College London, UK Lisa Hurley, NIHR Imperial Clinical Research Facility, Imperial College London, UK Matthew Pace: Peter Medawar Building for Pathogen Research, University of Oxford, UK Ane Ogbe: Peter Medawar Building for Pathogen Research, University of Oxford, UK Maryam Khan: Department of Infectious Disease, Imperial College London, UK Panagiota Zacharopoulou: Peter Medawar Building for Pathogen Research, University of Oxford, UK Helen Brown: Peter Medawar Building for Pathogen Research, University of Oxford, UK Euan Sutherland: Imperial College Clinical Trials Centre, Imperial College Healthcare NHS Trust, UK Hanna Box: Department of Infectious Disease, Imperial College London, UK Julie Fox: Harrison Wing, Guy’s and St Thomas Hospital NHS Foundation Trust, UK Steven Deeks: Department of Medicine, University of California, San Francisco, California 94110, USA Jill Horowitz: Laboratory of Molecular Immunology, The Rockefeller University, New York, USA Michel C. Nussenzweig: Laboratory of Molecular Immunology, The Rockefeller University, New York, USA Marina Caskey: Laboratory of Molecular Immunology, The Rockefeller University, New York, USA John Frater*: Peter Medawar Building for Pathogen Research, University of Oxford, UK Sarah Fidler*: Department of Infectious Disease, Imperial College London, UK The last two authors contributed equally to this manuscript.
Name and contact information for the trial sponsor {5b}	The trial is sponsored by Imperial College London as per the following contact details: Research Governance and Integrity Team Imperial College Academic Health Science Centre Room 221, Medical School Building, St Mary’s Campus, Norfolk Place, London W2 1PG Sponsor representative: Keith Boland Email: rgit.ctimp.team@imperial.ac.uk
Role of sponsor {5c}	This is an investigator initiated clinical trial. The funder and sponsor played a limited role in the design of the study, and will have no role in the collection, analysis, interpretation of data, decision to publish or preparation of future manuscript(s). These tasks are the responsibility of the trial steering committee {5d}. This manuscript was written by the authors {5a}

## Introduction

### Background and rationale {6a}

Antiretroviral therapy (ART) has dramatically improved survival for people living with HIV, preventing both disease progression and onward transmission. ART alone cannot cure HIV infection due to viral persistence in an inaccessible reservoir of latently infected cells [1]. Also, drawbacks to current ART include adherence requirements (usually to daily oral medication) and uncertain long-term safety profiles. These factors may contribute to the stigma and discrimination that limits social relationship, including vulnerability to legal action and a reduced quality of life, that are commonly faced by HIV-positive people [2]. The need for repeat drug refills and challenges to drug stock outs in low- and middle-income countries (LMIC) where most HIV-positive people live, also drives the need for alternative approaches to long-term HIV management.

Recent advances in long-acting broadly neutralising antibodies (bNAbs) to HIV *env* protein have shown promise in addressing these issues. Neutralising antibodies prevent pathogens from infecting host cells, by blocking virus entry through disrupting virus-receptor interaction, antibody-dependent cell-mediated cytotoxicity (ADCC), and effector cell-derived soluble factors inhibiting viral spread [3, 4]. There is an additional postulated mechanism whereby the antibodies induce HIV-specific CD8 mediated cellular immune responses similar to a vaccine effect [5]. Neutralising antibodies have been found in the course of natural infection in people with chronic HIV infection [6]. However, only 1% of them develop broad and very potent neutralising bNAb responses that can neutralise a wide range of HIV-1 variants due to the virus’s complex diversity and high mutation rate, and initial attempts to produce them ex vivo showed limited potency [7]. The development of high-throughput neutralisation assays and single-cell antibody cloning techniques have led to second-generation bNAbs with greater potency and breadth [8].

In a human trial conducted by Mendoza et al. [9], HIV-positive people on ART received a combination of 3BNC117 and 10-1074 just before and during analytical treatment interruption (ATI) (30 mg/kg of each antibody at 0, 3, and 6 weeks). The nine enrolled individuals with virus which appeared to be sensitive to both bNAbs maintained viral suppression for a median of 21 weeks, and none of the participants developed evidence of resistance to either bNAb. Two individuals who started ART during early HIV infection, maintained virus control until the end of study at 30 weeks. The combination bNAb infusion was well-tolerated with no serious adverse events.

The modification of the Fc receptor through the introduction of the LS mutation (substitution of Met428Leu and Asn 434Ser) was shown to extend serum antibody half-life [10, 11] for VRC01-LS compared to the wild-type VRC01 bNAb by up to 3- to 4-fold, suggesting the possibility of bi-annual or annual intravenous administrations for maintenance therapy for individuals where adherence to lifelong daily medications is challenging. A number of clinical trials are planned or in progress to investigate these bNAbs (Table 1) [12].

**Table 1:** Summary of current or future clinical trials involving bNAbs 3BNC117 or 10-1074 (including long-acting -LS versions)

Clinicaltrials.gov number	Intervention	Study design	PI, Sponsor	Start date	Primary endpoint
NCT03554408	bNAbs: 10-1074 LS and 3BNC-117 LS, alone and together	Phase I, dose escalation, first in man in HIV-negative and HIV-positive individuals. *n* = 75	Caskey, Rockefeller University, USA	Jun 2018	Safety and tolerability
NCT03619278	bNAb: 10-1074, Latency reversal agent (LRA): Romidepsin, Vaccine: HIVACAR01	Phase I/IIa randomised triple-blind placebo-controlled trial *n* = 12	PI: Garcia Cinca, IDIBAPS, Barcelona, Spain	Nov 2020	Safety
NCT03588715 BEAT-2	Peg-IFN-a2b, bNAbs: 3BNC117, 10-1074	Phase I randomised open-label trial *n* = 21	PI: Montaner, University of Pennsylvania, USA	Jun 2020	Safety Frequency of VL < 50 copies/ml at week 8 after ATI Innate activation
NCT03041012 eCLEAR	bNAb: 3BNC117, LRA: Romidepsin	Phase I open-label 4-arm randomised controlled trial *n* = 60	PI: Søgaard, Aarhus University Hospital, Denmark	Jan 2017	Time to VL < 20 copies/ml Quantification of proviral HIV reservoir
NCT03837756 TITAN	TLR9 agonist: Lefitolimod, bNAbs: 3BNC117, 10-1074	Phase II randomised placebo-controlled double-blinded study *n* = 48	PI: Søgaard, Aarhus University Hospital, Denmark	May 2019	Time to VL > 10 000 copies/ml × 3 or end of ATI
NCT04357821	Vaccines: IL-12 adjuvanted p24CE DNA prime, IL-12 adjuvanted DNA boost, MVA/HIV62B boost, bNAbs: VRC07-523LS, 10-1074, TLR9 agonist: Lefitolimod	Phase I/II Single group open-label combination intervention trial *n* = 20	PI: Deeks, University of California, USA	Aug 2020	Safety Proportion achieving post-treatment control
NCT04408963	bNAb: CAP256V2LS via intravenous or subcutaneous administration	Phase I Open-label trial *n* = 20	PI: Widge, National Institute of Health Clinical Centre, Maryland, USA	Mar 2021	Safety Tolerability
NCT03538626	bNAb: N6LS Recombinant human hyaluronidase (rHuPH20)	Phase I open-label dose-escalation trial *n* = 40	PI: Gaudinski, National Institute of Health Clinical Centre, Maryland, USA	Jun 2018	Safety Tolerability
NCT04173819	bNAbs: 3BNC117-LS-J 10-1074-LS-J	Phase I/II Randomised double-blinded placebo-controlled trial *n* = 225	PI: Caskey, Rockefeller University, USA	Jan 2019	Safety Pharmacokinetics
NCT04560569	Albuvirtide (long-acting fusion inhibitor) bNAb: 3BNC117	Phase II open-label non-randomised trial *n* = 20	PI: Yao Frontier Biotechnologies Inc., USA	Apr 2021	Proportion of participants with ≥0.5log reduction in HIV-1 VL from baseline to day 14
NCT03571204	bNAbs: 3BNC117, 10-1074	Phase I triple-blinded randomised placebo-controlled trial *n* = 50	PI: Sneller, National Institutes of Health Clinical Centre, Maryland, USA	Sep 2018	Safety Tolerability
NCT04250636	bNAbs: 3BNC117-LS, 10-1074-LS	Phase I open-label single arm study *n* = 10	PI: Caskey, Rockefeller University, USA	Aug 2020	Safety Pharmacokinetics Decline in HIV VL through week 4
NCT03719664	Albuvirtide (long-acting fusion inhibitor) bNAb: 3BNC117	Phase II open-label randomised controlled trial *n* = 80	PI: Yao Frontier Biotechnologies Inc., USA	Apr 2020	Proportion with HIV VL < 50 copies/ml
NCT03707977	bNAbs: VRC01LS, 10-1074	Phase I/II open-label non-randomised trial in children *n* = 40	PI: Shapiro, Kuritzkes, Licterfield National Institute of Allergy and Infectious Diseases, USA (Study in Botswana)	Jun 2019	Safety Proportion with HIV VL < 400 copies/ml Proportion with HIV VL < 40 copies/ml

ATIs are a recognised strategy for HIV research to understand the HIV reservoir dynamics and validate strategies for long-term ART-free viral suppression, until biomarkers to predict post-treatment viral control become available. Consensus recommendations for the ethical conduct of trials involving ATI were agreed by a panel of experts on HIV research in 2018 [13], and we further explore the benefits and risks of ATI trials in the discussion later in this manuscript.

In this paper, we describe the methods of the RIO trial. The RIO trial aims to investigate the use of a novel combination of long-acting broadly neutralising antibodies 3BNC117-LS and 10-1074-LS in participants who initiated ART early during primary HIV infection (PHI), in preventing HIV viral rebound after stopping ART for an extended period. We also describe the impact of the COVID-19 pandemic, the modifications to the trial protocol as a result, and the key role of community contribution throughout the study design, management, oversight, and adaptation to the COVID-19 pandemic measures.

### Objectives {7}

#### Primary objective

- To determine the efficacy of dual bNAb infusion of 10-1074-LS and 3BNC117-LS at sustaining virological control within 36 weeks following initial ATI compared with placebo infusion.

#### Secondary objectives

- To evaluate the safety of dual bNAb infusion of 10-1074-LS and 3BNC117-LS.
- To determine the role of viraemia at the time of bNAb administration in subsequent virological control.
- To determine the contribution of circulating bNAbs to virological control compared with a sustained impact following antibody elimination.
- To document the experiences and determine the attitudes of participants to the interventions and the treatment interruption.

#### Tertiary objective

- To determine the mechanisms by which bNAbs infusions can induce sustained virological control after stopping ART.

### Trial design {8}

**Fig. 1:**
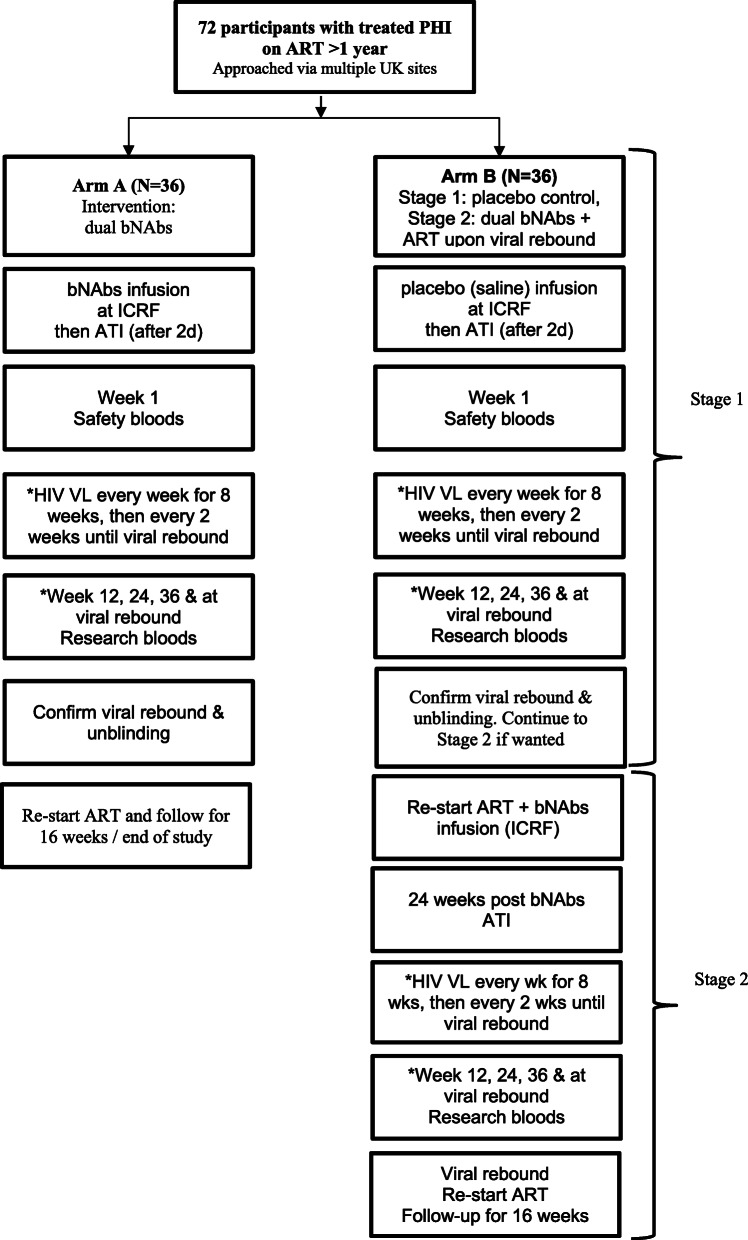
Summary of trial design

The RIO study is a randomised, placebo-controlled double-blind two-arm prospective phase II trial in two stages, summarised in Fig. 1. All parties blinded are listed later in this paper under ‘Who will be blinded {17a}’.

#### Stage 1—72 individuals

##### Arm A

- Recruit 36 individuals.
- bNAbs given once followed by ATI 2 days later.
- ATI stage up to viral rebound or end of study.
- Restart ART and follow-up for 16 weeks or to end of study.

##### Arm B

- Recruit 36 individuals.
- Placebo infusion given once followed by ATI 2 days later.
- 1st ATI stage up to viral rebound or end of study.

#### Stage 2—36 individuals from Stage 1 arm B

- Restart ART, with immediate bNAb infusion followed by washout period of 24 weeks.
- 2nd ATI up to viral rebound or end of study.
- Restart ART and follow-up for 16 weeks or to end of study

## Methods: participants, interventions, and outcomes

### Study setting {9}

The target study population include eligible and consenting people living with HIV who commenced ART during confirmed PHI and who have remained with viral suppression below the level of detection (< 50 copies HIV RNA/ml) for at least 1 year. Clinical care will be provided through the National Health Service (NHS). Research study visits will be undertaken at one of the clinical sites in accordance with the trial protocol, all within the UK. Clinical sites are listed in Appendix 1.

All intervention study visits (involving infusions of bNAbs or placebo) will take place at the NIHR Imperial Clinical Research Facility (ICRF) at Hammersmith Hospital, London, UK.

### Eligibility criteria {10}

#### Inclusion criteria

- Aged ≥ 18 to ≤ 60 years old at screening.
- Able to give informed written consent including consent to long-term follow-up.
- Willing and able to comply with visit schedule and provide blood sampling.
- Started ART within a maximum of 6 months of estimated date of primary HIV infection, based on one of the following six criteria:
  - Positive HIV-1 serology within a maximum of 24 weeks of a documented negative HIV-1 serology test result (can include point of care test (POCT) using blood for both tests). The estimated time of infection is taken as the midpoint between the dates of the negative HIV-1 serology or POCT test and positive HIV test at diagnosis, unless there was clear evidence for a transmission event, in which case that date will be taken.
  - The date of a positive p24 antigen result and a negative HIV antibody test.
  - The date of a negative HIV antibody test with either detectable HIV RNA or proviral DNA.
  - Recent infection testing algorithm (RITA) reported as ‘Incident’ confirming the HIV-1 antibody avidity is consistent with recent infection (within the preceding 16 weeks). The estimated date of infection is assumed to be 2 months prior to the date of the incident test result.
  - The date of a weakly reactive or equivocal 4th-generation HIV antibody antigen test.
  - The date of an equivocal or reactive antibody test with less than 4 bands on western blot.
- Stable on ART with suppressed undetectable HIV viral load (VL) ‘target not detected’ using local assays for ≥1 year.
- No evidence of viral insensitivity to either 10-1074 or 3BNC117 antibodies based on proviral sequencing algorithm.
- Hepatitis B virus (HBV) surface antigen or HBV DNA, hepatitis C virus (HCV) Ag or HCV RNA negative or HCV anti-core antibody negative.
- No significant comorbidities.
- Nadir CD4 > 350 cells/μL.
- Current CD4 count > 500 cells/μL or CD4:CD8 ratio > 1.
- On integrase inhibitor (INSTI) or boosted protease inhibitor (bPI)-based regimen at time of randomisation, if previously on non-nucleoside reverse transcriptase inhibitor (NNRTI) has switched at least 4 weeks prior to randomisation.
- Adequate haemoglobin (≥120 g/L for males, ≥110 g/L for females).
- Weight ≥ 50 kg.
- Females capable of becoming pregnant must agree to use hormonal contraception, intrauterine device, intrauterine hormone-releasing system, or to complete abstinence from at least 2 weeks before the first bNAb/placebo infusion and for 20 months after the last bNAb infusion.
- Participants must have been vaccinated against coronavirus (COVID-19), at least 4 weeks prior to enrolment.

#### Exclusion criteria

- Previous ischaemic heart disease (ST or non-ST myocardial infarction, QRISK3 10-year cardiovascular risk > 20%, stable angina, unstable angina, stroke)
- Any current or past history of malignancy, excluding squamous cell skin cancers.
- Concurrent opportunistic infection or other comorbidity or comorbidity likely to occur during the trial, e.g. malabsorption syndromes, autoimmune disease.
- Any contraindication to receipt of British HIV Association (BHIVA) recommended combination antiretrovirals [14].
- HTLV-1 co-infection
- SARS-Cov-2 infection confirmed by SARS-Cov-2 RT-PCR positive result from nasopharyngeal swab up to 72 h prior to randomisation/dosing visit.
- Individuals at high risk from severe COVID-19 disease who may be defined in accordance with National Health Service England guidance [15] as vulnerable and shielded (as per the view of participant’s physician)
- Current or planned systemic immunosuppressive therapy (inhaled or topical corticosteroids are allowed.)
- Participation in any other clinical trial of an experimental agent or any non-interventional study where additional blood draws are required; participation in an observational study is permitted.
- History of anaphylaxis or severe adverse reaction to antibody infusions, or hypersensitivity to 3BNC117-LS or 10-1074-LS or to or any constituent products or excipients
- Treatment with intravenous immunoglobulin or other monoclonal antibody treatments planned during the duration of the trial.
- Clinically significant abnormal blood test results at screening including:
  - Moderate to severe hepatic impairment as defined by significant liver impairment with evidence of advanced fibrosis or cirrhosis with decompensation.
  - Alanine transaminase (ALT) >5× upper limit of normal.
  - Estimated glomerular filtration rate < 60 ml/min/kg.
  - Urine protein creatinine ratio > 30 mg/mmol.
  - International normalised ratio (INR) > 1.5.
- Physical examination findings: Evidence of organ dysfunction or any clinically significant deviation from normal in physical examination and/or vital signs that the investigator believes is a preclusion from enrolment into the study.
- Active alcohol or substance use that, in the Investigator’s opinion, will prevent adequate adherence with study requirements.
- Insufficient venous access that will allow scheduled blood draws as per protocol.
- Concern regarding likelihood of participant not taking precautions to prevent HIV transmission during treatment interruption period.
- Pregnancy or breastfeeding

### Who will take informed consent? {26a}

Potential participants will be approached directly by their usual HIV clinician at their clinical site, or by investigators working on the HEATHER cohort study. The HEATHER study is an observational cohort of individuals with documented PHI who commenced on immediate ART within 3 months of HIV acquisition and are invited to have research blood samples taken at regular intervals timed with their regular clinic visits. The cohort has recruited over 350 individuals with treated PHI and acts as a platform for further research studies such as the RIO study (Approved by West Midlands – South Birmingham Research Ethics Committee reference 14/WM/1104).

Those interested in joining the study will be offered an appointment with a Good Clinical Practice (GCP) trained member of the research team. Individuals will then be informed of the details of study and invited to participate, with informed consent given by participants to research staff who are suitably qualified and have been authorised to do so by the CI/Principal investigator (PI) according to GCP guidelines.

### Additional consent provisions for collection and use of participant data and biological specimens {26b}

Consent for the use of blood samples in future research and to transfer samples outside of UK and EU countries will also be obtained.

The participant will be given a minimum of 24 h and the opportunity to question the Investigator, their GP, or other independent parties to decide whether they will participate in the study. Written Informed Consent will then be obtained by means of participant dated signature and dated signature of the person who presented and obtained the informed consent.

## Interventions

### Explanation for the choice of comparators {6b}

The RIO trial design aims to answer two important research questions;

1. To determine time to viral rebound following one infusion of dual bNAbs compared with placebo. This will inform the field to quantify the impact of the intervention on ART-free viral control. This is the first study to address this research question.
2. To determine if receiving dual bNAbs at the time of ART restart following an ATI can confer post ART viral control after 6 months of ART, at a point when it is anticipated that plasma concentration of bNAbs will be below therapeutic levels.

The second aspect of the trial will explore the scientific question as to whether the lasting impact of dual bNAb treatment affects immune function and whether the ‘vaccinal’ effect of stimulating HIV-specific CD8+ and NK cell activation sufficiently to contain viral replication is a true in vivo phenomenon. Additionally, such is the interest and support from the community representatives that they were very supportive of this two-step design as it enables all eligible interested study participants to receive bNAb.

Comparators for participants receiving the intervention in arm A are participants meeting the inclusion criteria who are randomised into arm B and receive a placebo infusion followed by subsequent ATI.

### Intervention description {11a}

#### Investigational Medicinal Products (IMP) - bNAbs

The RIO trial involves administration of two bNAbs: 10-1074-LS and 3BNC117-LS, and placebo infusions (0.9% sodium chloride solution). 3BNC117-LS is a recombinant, fully human monoclonal antibody (mAb) of the IgG1κ isotype that specifically binds to the CD4 binding site (CD4bs) within HIV-1 envelope gp-120.

10-1074-LS is a recombinant, fully monoclonal antibody of the IgG1λ isotype that specifically binds to the base of the V3 loop within the HIV external membrane glycoprotein, gp120.

The original antibodies 3BNC117 and 10-1074 were identified and cloned at the Rockefeller University from an HIV-positive individual. Both 3BNC117-LS and 10-1074-LS differ from the original parental antibodies by two amino acid substitutions which were introduced for the purpose of extending their biological half-life.

Placebo arm B will receive 0.9% sodium chloride (normal saline) infusion identical in appearance and volume to the bNAb infusions.

Investigator brochures (IB) for 10-1074-LS and 3BNC117-LS IB will be used in the trial.

Both bNAbs will be stored at 5 ± 3 °C, and each bNAb vial contains a nominal 150 mg/ml of bNAb protein in a 1.0-mL buffered solution.

#### bNAb dosage and administration

3BNC117-LS and 10-1074-LS will each be administered at a dose of 30 mg/kg and 10 mg/kg respectively. Prior to infusion, each IMP is diluted to a final volume of 100 mL in a single normal saline injection. Each IMP is then administered as a separate intravenous infusion over a period of approximately 30 min per bNAb. IMP infusion should be administered through a 0.2 or 0.22 micron in-line filter.

#### Antiretroviral treatment interruption

Individuals on triple ART with suppressed HIV VL will be enrolled into the study and receive either dual bNAb or placebo, 3 days later they will then stop ART. The date of ART stop is considered time 0 for time to viral rebound estimation. At the time of recruitment, all participants will be receiving ART according to current best practice and the BHIVA guidelines [14]. At the time of randomisation, participants’ ART regimen must include an INSTI or bPI; if on an NNRTI, they must be switched to an INSTI or bPI at least 4 weeks prior to randomisation [16]. The ART regimen after viral rebound should ideally include an INSTI or bPI. Other changes in ART outside the protocol-mandated ART interruption period, and any other concomitant medical therapy either at screening or during the study will be recorded.

##### Management of ATI

Following interruption of ART, all participants will have their CD4 count and plasma HIV VL monitored using routine NHS validated measurements. These will be at a minimum weekly for the first 8 weeks and then every 2 weeks until viral rebound has occurred and/or ART is recommenced (according to any of the specified restart reasons) based on the thresholds described below. Results will be available within 24 h of laboratory receipt of samples. Results of the laboratory HIV VL will be given to the participant by the research study team, using the participant’s pre-stated preferred method (text, email, telephone, or in-person consultation).

Any study participant wishing to attend for additional visits for VL monitoring may do so. In exceptional circumstances (i.e. positive for SARS-Cov-2 or self-isolating), if participants are unable to attend in person for study visit, home visit will be arranged for the collection of blood sample for HIV VL monitoring.

##### Criteria for restarting ART

1. Viral rebound confirmed by HIV VL measurement from venous blood:
  - Sustained HIV viral load ≥1000 copies/ml for 6 weeks +/− 7 days.
  - Is ≥100,000 copies/ml for two readings, 7 days apart +/− 7 days.
  - Instances outside of the above two categories but are close to these definitions and necessitate ART restart for clinical reasons. These instances will be individually reviewed by an independent panel (Endpoint Adjudication Committee) to confirm if they meet the definition for viral rebound.
2. CD4 count drops to less than 350 cells/μL.
3. Clinical symptoms attributable to ATI.
4. Participant preference.
5. Major concerns over risk mitigation for HIV transmission.
6. Positive for COVID-19 (SARS-CoV-2 PCR positive) during ATI.

**Fig. 2:**
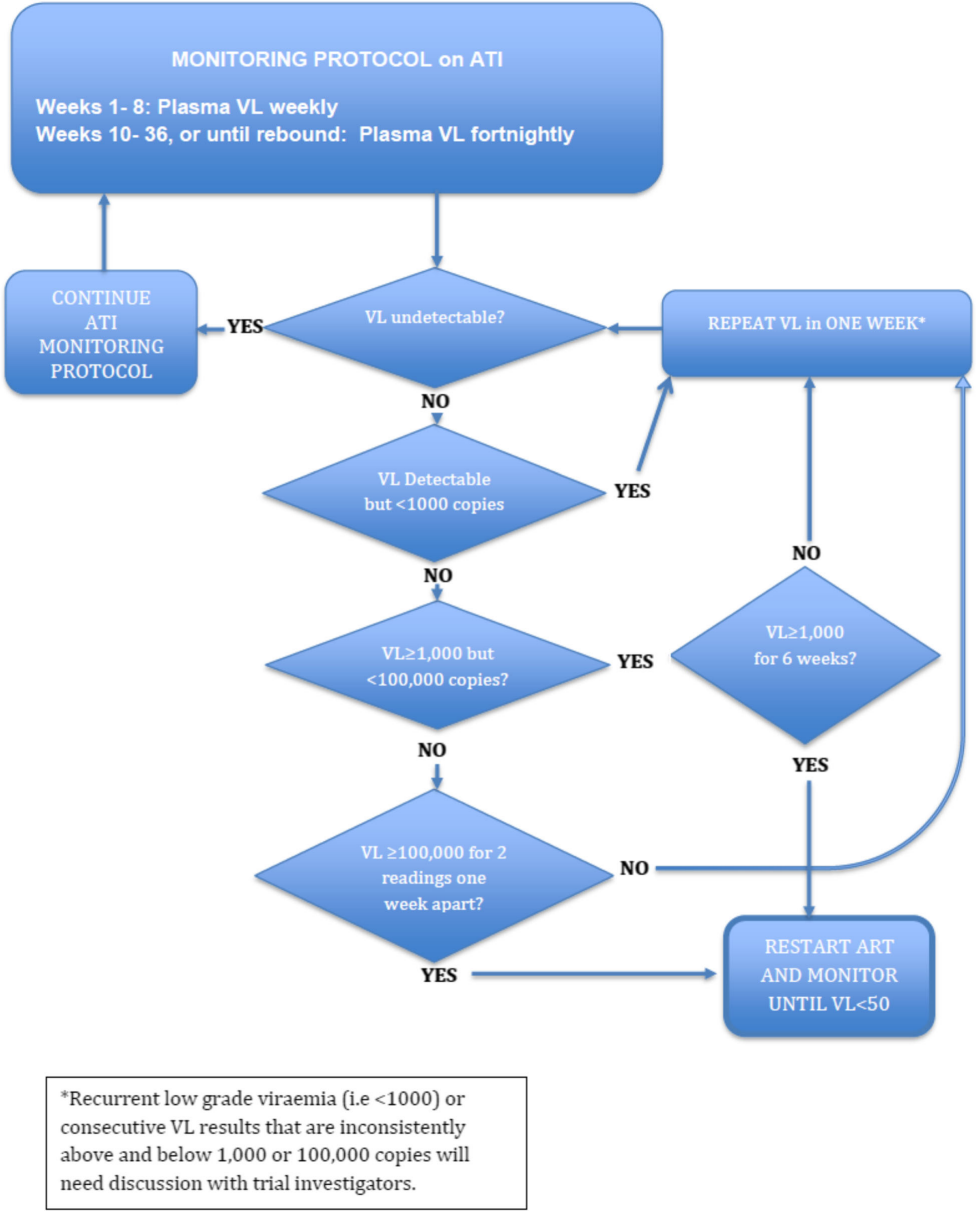
Monitoring protocol on analytical treatment interruption

The clinical decision algorithm for management of detectable viral load and viral rebound is summarised in Fig. 2. In addition,

- At any VL threshold, if the participant prefers, ART can be restarted.
- It is possible that some participants will have consecutive VL that are repeatedly above and below 1000 copies/ml and therefore do not formally meet criteria for rebound, and yet have evidence of sustained viraemia. In these cases, the Endpoint Adjudication Committee will consider these instances and confirm whether they can be defined as viral rebound.
- If there is a confirmed drop in CD4 count to < 350 or in the presence of symptoms that maybe attributed to the ATI, ART will be recommenced.

- Restarting ART
  - Participants will be followed up at weeks 2, 4, 8, and 12 (with viral load testing) after reinitiation of ART or until their viral load becomes undetectable; they will be subsequently followed up until the end of the study. An additional venous blood sample (100 ml) will be taken at 16 weeks after restarting ART for research purposes.
  - Any study participant who wishes to restart ART will be allowed to do so at any time during the ATI. Upon ART restart, the visit schedule will be as described above for participants restarting ART.

Participants who do not experience HIV rebound viraemia off ART within 36 weeks will continue to remain off therapy for the duration of the study or until viral rebound occur. At the end of the study, all participants will be recommended to restart ART irrespective of HIV viral load in accordance with BHIVA guidelines.

Participants will be clearly informed of the potential increased risk of onward transmission during the period off therapy, when there is a possibility of detectable viraemia. Participants will be advised to use condoms for all sexual contacts and be aware of the availability and access to post-exposure prophylaxis (PEP) for any HIV-negative partners should an exposure occur or if a condom breaks, particularly during ATI. In addition, any sexual partners of the participants wishing to discuss and access HIV pre-exposure prophylaxis (PrEP) will be invited to attend the RIO research team for HIV testing and where appropriate will be offered PrEP and be monitored through NHS sexual health clinics.

With the participant’s consent, their routine HIV clinician and general practitioner (GP) will be informed of their participation in the study.

### Criteria for discontinuing or modifying allocated interventions {11b}

#### bNAb dose modification for toxicity.

Rates of infusion of the bNab may be adjusted in the event of an adverse reaction. If this persists, the infusion will be halted.

### Strategies to improve adherence to interventions {11c}

#### Adherence to ART

ART treatment compliance will be the responsibility of each study participant and their routine HIV physician. Investigators will promote compliance by providing instruction for participants to take ART exactly as prescribed during the course of the trial. Participant compliance to ART or remaining off ART will be closely monitored throughout the trial through regular HIV viral load monitoring. Blood measurement of ART levels will be undertaken retrospectively to ensure that for evaluation of the primary endpoint individuals with controlled viraemia are not on any ART drugs.

#### Adherence to follow-up during ATI period

HIV viraemia will be monitored during ATI as described above.

If a participant does not attend a scheduled visit, the study clinical team must make reasonable attempts to contact the participant to remind them to attend as soon as possible.

If a further visit is missed, participants will be reminded of the risks to their own health and of onward viral transmission, and encouraged to restart ART. The use of condoms, PEP, and PrEP for HIV-negative partners to prevent onward transmission of HIV will be discussed and all HIV-negative partners will be linked into NHS PrEP services.

If a participant has missed study visits in a 4-week period, a letter will be sent to the participant, their routine HIV clinician and GP. A recommendation to restart ART would be given to these patients not attending follow-up visits during the ATI period, and they will continue to be invited to attend study follow-up visits until the end of the trial.

### Relevant concomitant care permitted or prohibited during the trial {11d}

#### Concomitant care prohibited during the trial.

Treatment with intravenous immunoglobulin or other monoclonal antibody treatments is prohibited for the duration of participation in the trial.

#### Concomitant care permitted during the trial.

Monitoring and medical care will be provided as per standard care, except during ATI where participants’ medical care will be provided as described above.

### COVID-19 pandemic measures

All study visits will take place in COVID-19 free areas and participants will be provided appropriate personal protective equipment (PPE), and trial procedures conducted as per current COVID-19 guidelines/practices [17] until such guidelines/practices are no longer applicable or relevant.

A protocol amendment was introduced to the eligibility criteria that participants would only be included if they had received at least one dose of a SARS-CoV-2 vaccine. Single-dose vaccine efficacy of the BNT1626b2 mRNA vaccine (Pfizer BioNTech) and ChAdOx1 nCoV-19 vaccine (AZD1222) has been estimated to be high at 89% and 73% respectively [18], and contributed to the rationale to offer enrollment to participants while awaiting their second dose in the vaccine schedule recommended by the UK joint committee on vaccination and immunisation [19]. In a HIV-positive cohort, there were no differences in both humoral and cell-mediated immune responses produced by the ChAdOx1 nCoV-19 vaccine compared to a HIV-negative cohort [20].

If participants exhibit SARS-CoV-2 symptoms during ATI or any other time during the trial, participants will be tested for SARS-CoV-2. If PCR positive for SARS-CoV-2, individuals who have interrupted ART will be asked to restart ART. A review with the CI, PI, participant, and their clinical team, on a case-by-case basis, will evaluate whether repeat ATI will be undertaken, after clearance of SARS-CoV-2 and resolution of symptoms. All study participants will have access to current/ongoing recommended NHS COVID-19 treatment.

### Provisions for post-trial care {30}

At the end of the study, all participants will be recommended to restart ART irrespective of HIV VL in accordance with BHIVA guidelines.

Participants will be referred to their routine HIV clinic for ongoing care and follow-up post-trial.

Admission to hospital if required will be provided to all study participants through NHS clinical services.

### Outcomes {12}

The primary endpoint is the comparison of time to viral rebound within 36 weeks after initial ATI, during Stage 1, summarised by hazard ratios.

Secondary endpoints, for both stages 1 and 2, are:

1. Safety defined as the number and types of adverse events (AEs) and serious adverse events (SAEs) by group
2. Length of time undetectable in days following ATI in the absence of detectable ART (comparing arm A vs B and arm B Stage 1 ATI vs Stage 2 ATI), summarised by mean.
3. CD4 T cell counts and CD4:CD8 ratios at weeks 12-, 24-, 36-, and 48-week post randomisation, and 12-weekly until the end of study participation by arm, summarised by median.
4. Percentage of participants with undetectable VL at week 12, 24, 36, and 48 post randomisation (Stage 1; arm A vs B) and then for arm B participants post second ATI
5. Quantitation of proviral HIV DNA and cell-associated RNA at baseline and following viral rebound and resuppression on ART by arm.
6. Duration of remission analysed under multiple alternate definitions (e.g. VL < 40 copies/mL, < 400 copies/mL, < 1000 mL), comparing arm A vs B and arm B Stage 1 ATI vs Stage 2 ATI, summarised by mean.
7. Time to restarting ART after start of ATI, comparing arm A vs B and arm B Stage 1 ATI vs Stage 2 ATI, summarised by median.
8. Time to undetectable HIV VL after restarting ART, comparing arm A vs B and arm B Stage 1 ATI vs Stage 2 ATI, summarised by median.
9. The proportion of participants with detectable ART levels in blood during ATI, at weeks 12, 24, 36, and 48 post randomisation, by arm.
10. bNAb concentration in blood at dosing, weeks 2, 4, 8, 12, 24, 36, and 48, time of viral rebound, and end of study, summarised by mean.
11. Proportion and description of bNAb resistance/sensitivity at pre and post infusion time-points, by arm.
12. Descriptive changes in reported HIV Quality of Life measures at baseline, 2 weeks after infusion, and at the end of study [21].

Tertiary or exploratory endpoints, for both stages 1 and 2, will include:

1. HIV-specific (humoral and cell-mediated) and innate immune responses
2. Immune phenotyping and activation/exhaustion
3. Host gene expression
4. Viral sequence and integration site analysis
5. Measures of the HIV viral reservoir in CD4+ T cells.

### Participant timeline {13}

The study visit schedules are summarised in Tables 2 and 3.

**Table 2:**
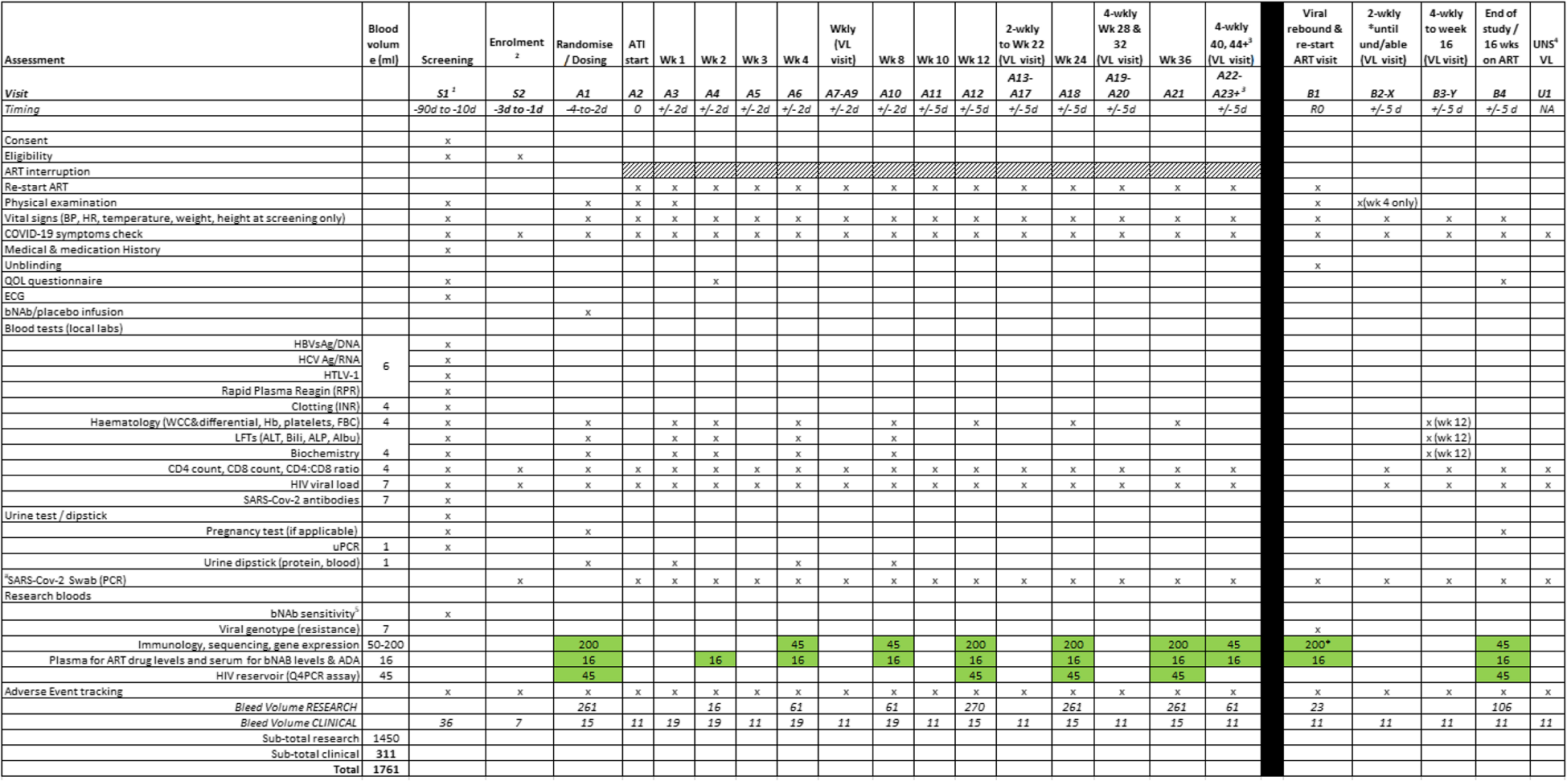
Trial assessment schedule for Stage 1 (both arms A and B)

1. Visit prefix nomenclature: Screening ‘S’; During ATI ‘A’; After Rebound ‘B’ After Second Rebound ‘C’

1. Screening and Enrolment visit may be combined into one visit where possible

2. 4-weekly visits repeated until viral rebound occurs

3. UNS: patients may request an additional ‘unscheduled’ visit to have HIV VL measured any time while still unsuppressed

4. bNAb sensitivity visit at screening not required if done as part of the HEATHER study

5. Arm B/Placebo participants (if they agree) will move to Stage 2 after viral rebound to continue with the study visits, B1 visit procedure will be conducted as per Stage 2 schedule, otherwise participants will follow Stage 1 visit schedule until the end of the study

6. ^#^SARS-Cov-2 PCR will be conducted up to 72 h prior to randomisation/dosing visit and for the rest of the visits PCR will only be performed if participants exhibit COVID-19 (SARS-Cov-2) symptoms, however SARS-CoV-2 PCR testing can be offered to any asymptomatic participant attending at any study visit in accordance with local NHS guidelines, and participant or clinical team choice.

7. ATI Start visit (A2) can be conducted as an onsite or telephone visit if participant prefers

8. Arm B: Research bloods can be taken at the viral rebound visit or the Stage 2 dosing visit – not both visits.

9. SARS-Cov-2 antibodies blood test at screening is an optional test and is not used to determine if the patient is eligible for the study

**Table 3:**
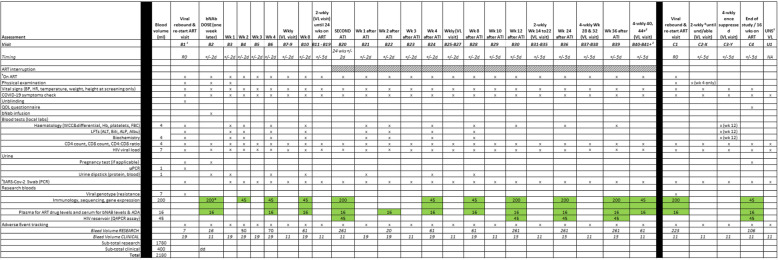
Trial assessment schedule for Stage 2 (arm B only)

1. Visit prefix nomenclature: Screening ‘S’; During TI ‘A’; After First Rebound ‘B’; After Second Rebound ‘C’

2. 4-weekly visits repeated until viral rebound occurs

3. UNS: patients may request an additional ‘unscheduled’ visit to have HIV VL measured any time while still unsuppressed

4. ^#^SARS-Cov-2 PCR will be conducted up to 72 h prior to ATI visit (B20) and for the rest of the visits PCR will only be performed if participants exhibit COVID-19 (SARS-Cov-2) symptoms

5. Visit B1 can be conducted as an onsite or telephone visit for arm B participants. The safety bloods and physical examination can be conducted at the confirmatory viral rebound visit if participant chooses B1 to be a telephone visit.

6. Research bloods can be taken at the dosing visit or the viral rebound visit at Stage 1—not at both visits

7. Visits B11-19 can be 4-weekly visits if participant has had two previous undetectable viral load while on ART

### Sample size {14}

#### Sample size and power considerations

Stage 1—arm A compared to arm B

Underlying viral rebound rates at 36 weeks of 90% and 55% in the control and intervention groups respectively (i.e. 10% and 45% free from rebound by 36 weeks; hazard ratio 0.35) will be detected with 90% power using a two-sided log-rank test at the 5% significance level provided there are 31 participants per arm and no loss to follow-up or return to ART treatment prior to 36-week primary outcome. We propose to recruit 72 participants in order to provide an allowance of approximately 10% return to ART treatment, and loss to follow-up, prior to the earlier of either 36 weeks or a primary event.

Stage 2—arm B only

For patients in arm B, we will look at the within-person difference in ‘length of time undetectable (in days)’ between first and second period. Assuming a standard deviation of 14 days and a conservative correlation between the number of days in the first and second period of 0.7, 36 patients will allow detecting a difference of 6.6 days between the two periods with 90% power and two-sided alpha 0.05, including 15% dropout rate.

### Recruitment {15}

The study population comprises participants with confirmed primary HIV infection who started ART within 6 months of confirmed primary HIV infection, and who have remained on suppressive ART without interruption for at least 12 months. The HEATHER study provides a pre-existing cohort of participants whom will be invited to participate if eligible. In addition, interested participants otherwise eligible but not enrolled into the HEATHER cohort may also be enrolled.

Eligible consenting participants who have not previously been enrolled into the HEATHER study, but fulfil the RIO eligibility criteria, may also be included and clinicians from participating clinical sites will be asked to identify and discuss their potential participation to the RIO trial. Interested and eligible participants will be referred to research staff at the clinical site for recruitment.

A website was created for RIO, this will include information on the eligibility criteria and participants are able to contact trial staff if interested to take part in the study (https://riotrial.org/)

## Assignment of interventions: allocation

### Sequence generation {16a}

Randomisation and unblinding will be carried out using a web-based randomisation and Electronic Data Capture (EDC) system, called Openclinica. Randomisation will not be stratified but will be blocked to ensure equal allocation to each arm.

Stage 2 will be unblinded.

### Concealment mechanism {16b}

The study will initially be double-blinded to study arm. Participants will be randomly allocated to initial treatment with bNAbs or placebo control then subsequent bNAb in a 1:1 ratio. Participants will receive blinded, identical-looking infusions at the initial dosing visit. The second infusion visit for arm B will be unblinded.

Each participant will be assigned a unique trial ID which is linked to the treatment allocation for Stages 1 and 2 of the trial. With the exception of planned unblinding prior to commencing the second infusion in arm B, the treatment code must not be broken. Exceptions include medical emergencies when the appropriate management of the participant necessitates knowledge of the treatment, or if expedited reporting of an unexpected and related SAE to the Research Ethics Committee (REC) and Medicines and Healthcare products Regulatory Agency (MHRA) is required.

### Implementation {16c}

The research team at each clinical site responsible for the enrolment of participants and follow-up procedures except the administration of IMP or placebo will be blinded to study arm.

The study monitor and ICRF team (pharmacist, nurses, and doctors) will be the only people unblinded to study arm. They will be responsible for storing, dispensing, preparing, and delivering the infusion and placebo at the randomisation visit and will allocate and administer the IMP or placebo to participants.

## Assignment of interventions: Blinding

### Who will be blinded {17a}

Apart from ICRF research staff who will administer either the IMP or placebo, all trial participants, care providers, outcomes assessors, and data analysts will be blinded to the study arm allocation of each participant for the duration of Stage 1 of the trial.

ICRF research staff will be blinded to outcomes collection and analysis.

The Independent Data Monitoring Committee (IDMC) and study statistician preparing the IDMC report will require access to fully unblinded data.

### Procedure for unblinding if needed {17b}

#### Emergency unblinding

The trial EDC system will include an automated unblinding facility, in case unblinding is required. In the event that emergency unblinding of an individual participant is required, authorised staff (as documented on the delegation log) will follow trial procedures to unblind the participant in question and proceed with expedited reporting if required.

Investigators are encouraged to discuss the need for emergency unblinding with the Sponsor / CI (or designee) if the circumstances permit such a discussion. If discussion prior to emergency unblinding cannot take place, the coordination centre should be informed afterwards.

#### Unblinding following viral rebound

Participants in both study arms will be unblinded once viral rebound has been confirmed.

Participants will attend the research clinic, and the local study team will unblind the participant, discuss the arm the patient had been assigned to, and explain next steps, i.e. arm A to restart ART immediately and continue study visits, and arm B to restart ART + bNAb if they wish to continue with the study.

Unblinding will occur using the automated facility within the Openclinica system.

#### Unblinding for statistical analysis

When performing safety and interim analyses, the integrity of trial blind should be maintained, with the exception of the IDMC who will have access to fully unblinded data. The Study Statistician preparing the IDMC report will also require access to fully unblinded data in order to prepare the report. Unblinding in these cases should take place in accordance with Imperial Clinical Trials Unit (ICTU) Standard Operating Procedures (SOP) and documented accordingly.

### Data collection and management

### Plans for assessment and collection of outcomes {18a}

#### Screening tests

The following blood tests will be performed at screening to confirm eligibility, tests will be analysed via local laboratories:

- HBV surface antigen or HBV PCR
- HCV PCR or core antigen
- HTLV-1
- Serologic tests for syphilis: rapid plasma reagin (RPR)
- Liver function tests (LFTs): serum ALT, bilirubin, aspartate transaminase (AST), albumin, alkaline phosphatase (ALP)
- Biochemistry: serum sodium, potassium, phosphate, calcium, urea, creatinine, glucose, lipids, and cholesterol levels
- Haematology: haemoglobulin, white cell count and differential, platelets, clotting (INR), prothrombin time, activated partial thromboplastin time and fibrinogen, CD4 count, CD8 count, CD4:CD8 ratio
- HIV VL
- Blood samples will be collected at screening to measure SARS-Cov-2 antibody level.

A urine sample will also be taken at screening to assess the following:

- Urine protein creatinine ratio (uPCR)
- For female participants, a urine dipstick pregnancy test will be performed

A swab sample will also be collected at screening to determine the eligibility and will be assessed via local laboratories:

- PCR to detect SARS-Cov-2

#### Screening bNAb sensitivity:

In order to be eligible for recruitment to RIO, candidates need to assessed to be sensitive to both bNAbs. To assess sensitivity, viral sequences in blood samples from candidates will be screened prior to their recruitment to the trial.

Proviral HIV DNA is extracted from peripheral blood mononuclear cells (~ 20 × 10^6^) and HIV Env genes from single proviruses are amplified using the single genome amplification assay, which is based on limiting dilution nested polymerase chain reaction. A minimum of 20 Env genes per candidate will need to be successfully sequenced using Illumina technology to confer a final result. A computational algorithm will be used to determine the sensitivity of all sequences from each candidate, by checking specific residue positions on the protein chain [22].

Participants with viral sequences that appear to be resistant to one or both bNAbs will not be eligible to participate in RIO.

#### Blood tests after screening:

The following blood tests will be repeated as indicated; tests will be analysed via local laboratories:

- LFTs (*weeks 1, 2, 4, 8, fortnightly till viral rebound*)
- Biochemistry: (*Stage 1: screening visit, dosing visit, week1, viral rebound visit and 12 weeks post-viral rebound. Stage 2 visits include: week 1, week 2, and 12 weeks after viral rebound*)
- *Full blood count (FBC) as per visit schedule*, CD4 count, CD8 count, CD4:CD8 ratio, and HIV VL (*all visits*)

#### Urine test after screening

At IMP/placebo dosing and weeks 1, 4, and 8, a urine dipstick test will be performed to check protein and blood.

For female participants, the pregnancy test will be repeated prior to dosing with IMP/placebo and again at the end of the study.

#### SARS-CoV-2 symptom checks and PCR test

Coronavirus symptoms screening will be performed at all visits. Swab samples (SARS-Cov-2 PCR) will be taken at screening and repeated only if symptoms of COVID-19 disease are reported throughout the trial follow-up.

Participants positive for SARS-Cov-2 at screening visit will be re-screened (swab sample for SARS-Cov-2 PCR), once fully recovered from COVID-19 illness and wish to join or re-join the study protocol.

#### Viral load and CD4 count monitoring

This is described under interventions and in Fig. 2.

#### Exploratory / research samples:

Additional blood samples will be taken from consenting participants over the course of the trial for research purposes to help understand the mechanisms underpinning the trial outcomes. While the plasma load is undetectable, every 2 weeks, additional research samples (four 9 ml acid-citrate-dextrose (ACD) tubes) of blood will be taken for research analyses.

Analysis of research samples will be carried out at the research laboratories at the University of Oxford in the UK and Rockefeller University in the USA.

The details of the bleeds and volumes are given in the schedules in Tables 2 and 3. The main assays to be undertaken are detailed in Table 4.

**Table 4:** Summary of key research assays

Assay	Sample	Purpose	Time-point
T cell immunology			• Pre-ART where available • On ART • Pre-bNAb • Fortnightly during ATI • Following viral suppression
HIV-specific T cell intracellular cytokine staining for CD4 and CD8 phenotypic and functional responses	PBMC	Screen CD4 and CD8 T cell responses to HIV in response to bNAbs	
HIV ELISpots: γ-interferon responses with peptides targeted according to ICS responses	PBMC	Higher resolution analyses of specific responses to peptide level and how they are impacted by intervention	
CD8 tetramer responses targeted to specific peptides	PBMC	Further in-depth resolution of CD8 T cell functionality at cellular level	
HLA Class I and II typing (4-digit)	PBMC	Profiling of HLA alleles to shape immune responses and confer advantage/disadvantages	
T cell killing assays	PBMC	Functional killing assay to augment intracellular cytokine staining data	
Natural Killer (NK) cell responses			
Flow cytometry for NK cell phenotype and function	PBMC	Characterisation of NK function and profile in response to bNAbs	
ADCC and NK cell killing	PBMC	Functional assessment of bNAbs using characterised killing protocols	
NK-like population analyses (flow cytometry)	PBMC	High resolution characterisation of cells that fall between the innate and adaptive responses	
Virology			
Next-generation sequencing (NGS) single genome amplification (SGA) of HIV ENV: bNAb sensitivity	PBMC	Env sequencing to screen for bNAb sensitivity	Screening
Full length HIV sequencing	Plasma CD4 T cells	Full length haplotype analysis of viral and proviral populations pre and post intervention to determine correlations with response	• Pre-ART (virus) where available (HEATHER cohort) • On ART, and pre-ATI (provirus) • Rebound (virus) • Post resuppression (provirus)
HIV Integration site analysis	CD4 T cells	Understanding of clonality of viral reservoir and relationship of integration sites to reactivation potential and susceptibility to inhibition	Pre-ATI
HIV DNA and cell-associated RNA quantitation	CD4 T cells	Standard molecular analyses of proviral and HIV transcript quantitation, both surrogate markers of persistent infection and biomarkers of remission and rebound	• Pre-ART where available (HEATHER cohort) • On ART • Pre-bNAb • Pre-ATI • Post resuppression
Host genomics			
Transcriptome profiling* *Initially bulk, but with single-cell 10× resolution dependent on results	CD4 and CD8 T cells NK cells	Full host genomic analyses at bulk and single cell (likely tetramer driven for CD8 cells) to understand host determinants of outcomes	• Pre-ART where available (HEATHER cohort) • On ART • Pre- ATI
Pharmacokinetics and anti-drug antibodies			
bNAb pharmacokinetics Quantitation of ‘anti-drug antibodies’ (ADA)	Plasma	To determine bNAb plasma concentrations To determine development of inhibitory antibodies against bNAbs	• Post bNAb infusion

#### Peripheral blood mononuclear cells (PBMC) and plasma samples

Blood samples (between 50 and 200 ml depending on the time-point) for the collection of PBMCs and plasma will be taken to evaluate the parameters detailed in Table 4.

For arm A, samples will be collected according to the schedules in Table 2 at the following time-points: screening, dosing visit, week 8, 24, and 36 after ATI, at viral rebound on restarting ART, following resuppression 16 weeks after ART restart. For participants who maintain undetectable VL off ART after 36 weeks, a 50-ml blood sample will be taken fortnightly until rebound or the end of the study.

For arm B, samples will be collected according to the schedules in Table 3 at the following time-points: screening, dosing visit, weeks 8, 24, and 36 after ATI, at viral rebound on restarting ART. For participants who maintain undetectable VL off ART after 36 weeks, a 50-ml blood sample will be taken fortnightly until rebound or the end of the study.

Then, bloods will be taken again at bNAb dosing (1 week after ART restart), at start of 2nd ATI 24 weeks later, 8 weeks after start of 2nd ATI, and then fortnightly from 24 weeks after the 2nd ATI until rebound or the end of the study. Following rebound and ART restart, bloods will be taken at restart and following resuppression 16 weeks after ART restart.

#### Quality of life (HIV PROM)

A participant-reported outcome measure (PROM) will be completed at baseline, 2 weeks after commencing the ATI and then at the end of the study to evaluate quality of life of participants. The PROM to be used has been developed by Harding et al. [14], is patient-centred, and enables multi-dimensional measurement of health-related quality of life for people with HIV.

### Plans to promote participant retention and complete follow-up {18b}

Participants will be reimbursed for their time and travel costs, £120 for the randomisation visit (lasting up to a full day), and £25 for all other visits.

#### Discontinuation of study treatment or study withdrawal

- Participants may discontinue study treatment for the following reasons:
- At the request of the participant
- Adverse event / serious adverse event
- Allergic reaction to the investigational medicinal product
- Withdrawal from the study (defined as discontinuation of study treatment and study procedures) for the following reasons:
- Participant decision
- Loss to follow-up
- Procedures for withdrawal from the study
- If a participant permanently discontinues the trial intervention, they will be invited to continue to attend trial visits if possible, to allow for collection of key outcomes and safety data.
- If a participant withdraws from trial procedures, an assessment must be made as to whether trial data and samples collected to date can be retained and analysed for the trial. If the participant does not agree for data and samples collected to be retained, the samples must be destroyed, and data excluded from the analyses.
- Participants who have discontinued the trial intervention and/or have withdrawn from the trial will not be replaced, as the sample size for Stage 1 allows for potential loss to follow-up.
- Data management {19}

#### Source data

Source documents include original documents related to the trial, to medical treatment and to the history of the participant, and adequate source documentation must be maintained at participating sites to allow reliable verification and validation of the trial data. What constitutes source data for this trial will be outlined in the trial monitoring plan (Supplement file 1).

#### Language

CRFs will be in English. Generic names for concomitant medications should be recorded in the CRF wherever possible. All written material to be used by participants must use vocabulary that is clearly understood and be in the language appropriate for the study site.

#### Database

Trial data will be collected on an electronic case report form (eCRF). Data is entered into the EDC system by trained site personnel. All data recorded in the eCRF will be signed off by the Investigator or his/her appropriate designee. All changes made following initial submission of data will have an electronic audit trail with a date. Specific instructions and further details will be outlined in the study-specific eCRF manual.

#### Data collection

The principal means of data collection from participant visits will be Electronic Data Capture (EDC) via the internet using the Openclinica database. Data will include demographics, vital signs (blood pressure, heart rate, temperature, weight and height at screening only), physical examination (including COVID-19 symptom checks), blood test results, and questionnaire data.

Details of procedures for eCRF completion will be provided in a study-specific manual.

#### Documentation and data storage

The investigator must retain essential documents until notified by the Sponsor, and for at least 10 years after study completion. Participant files and other source data (including copies of protocols, CRFs, original reports of test results, IMP dispensing logs, correspondence, records of informed consent, and other documents pertaining to the conduct of the study) must be retained. Documents should be stored in such a way that they can be accessed/data retrieved at a later date. Consideration should be given to security and environmental risks.

No study document will be destroyed without prior written agreement between the Sponsor and the investigator. Should the investigator wish to assign the study records to another party or move them to another location, written agreement must be obtained from the Sponsor.

#### Archiving

All trial documentation, including that held at participating sites and the trial coordinating centre, will be archived for a minimum of 10 years following the end of the study.

#### Data sharing

Individual de-identified participant data collected during the trial (including data dictionaries) will be available to share after publication. Data will be made available to researchers who provide a methodologically sound proposal, to achieve aims in the approved proposal. Proposals/requests should be directed to the Chief Investigator and requestors will be asked to sign a data access agreement. Data sharing requests will be shared with IDMC and TSC for their review and approval, respectively. In the event where no TSC or IDMC is available and the study is still active, the request is to be reviewed by the Head of Statistics, Director of Operations, and the Quality Assurance Manager.

### Confidentiality {27}

The investigator must ensure that the participant’s confidentiality is maintained. On the CRF or other documents submitted to the Sponsors, participants will be identified by a participant ID number only. Documents that are not submitted to the Sponsor (e.g., signed informed consent form) should be kept in a strictly confidential file by the investigator.

The investigator shall permit direct access to participants’ records and source documents for the purposes of monitoring, auditing, or inspection by the Sponsor, authorised representatives of the Sponsor, Regulatory Authorities, and RECs.

The investigators and study site staff will comply with the requirements of the Data Protection Act 2018 concerning the collection, storage, processing, and disclosure of personal information and will uphold the Act’s core principles.

It is the investigator’s responsibility to inform the participant’s routine doctor by letter that the participant is taking part in the study provided the participant agrees to this, and information to this effect is included in the Participant Information Sheet and Informed Consent. The participant’s GP will also be informed, providing participants agree to this. A copy of the letter should be filed in the Investigator Site File.

### Plans for collection, laboratory evaluation, and storage of biological specimens for genetic or molecular analysis in this trial/future use {33}

Samples will be collected according to the schedules in Tables 2 and 3 and analysed as described in Table 4. They will be stored in accordance with local laboratory practice, compliant to the Human Tissue Act 2004.

## Statistical methods

### Statistical methods for primary and secondary outcomes {20a}

Statistical methods for analysis of the study endpoints are described below. A separate detailed Statistical Analysis Plan (SAP) will be prepared and finalised prior to database lock. This will contain the rationale for the methods chosen and the assessment of their assumptions. It will include pre-specifying the handling of covariates and missing data and per protocol analyses. The per-protocol (PP) population will remove participants that are non-compliant in their treatment regimen. This will consist of the following: any participant that fails to successfully receive infusion (placebo or bNAb), any participant that fails to adhere to ATI, as established by detection of ART within blood sample, any participant that is lost-to-follow-up/withdrawn, any participant that fails to either (a) complete the 36-week follow-up period or (b) register viral rebound with the EAC. ITT population will include all participants randomised into the study according to their allocated arm at randomisation, regardless of treatment received. Changes to the plan will be re-approved by trial oversight committees.

Any deviations from the SAP will be documented and signed off by the statisticians and CI and filed in the statistics section of the Trial Master File (TMF) which will be merged with the main TMF at the end of the study.

- Primary endpoint analysis
- All the analyses will be on an intention-to-treat (ITT) principle and all statistical tests will be two-tailed with a 5% significance level. All participants screened eligible and enrolled in the trial will be included in the ITT analyses.
- The primary endpoint will be analysed using a time to event approach and differences will be tested using Cox proportional hazards models with censoring applied (as defined within the SAP). Kaplan-Meier curves detailing the survival function will also be presented.
- Secondary outcomes will be compared based on data type. Time to event outcomes will be assessed using the same Cox proportional hazards models described above. Paired data in Stage 2 will be assessed using a paired *t*-test (or alternate paired approach) and chi-squared / Fisher exact testing used for categorical variables. Further details to be provided in the SAP.
- Safety analysis
- Safety outcomes will be reported as unadjusted participant proportions and rates within and between arms with 95% confidence intervals using exact methods where appropriate.

An independent Endpoint Adjudication Committee will be set up to evaluate and review confirmed time of viral rebound in retrospect prior to the primary analysis. The committee will review all viral load data (blinded to study arm) and determine the date of confirmed viral load rebound. Any participant recommencing ART prior to viral rebound will also be reviewed by the committee as a potential endpoint. If established as not an endpoint, these participants will be censored at time of ART restart for analysis purposes.

### Interim analyses {21b}

No formal interim analysis is planned for the study outside of the reports compiled and presented to the IDMC. The reports will cover both safety and potential efficacy of study treatment. The primary analysis, comparing time to viral rebound between placebo and dual bNAbs, will be carried out once Stage 1 is complete. A second set of analysis exploring arm B exclusively will be carried out upon completion of Stage 2.

### Methods for additional analyses (e.g. subgroup analyses) {20b}

Alongside the intention-to-treat analysis a per-protocol (PP) analysis will be carried out for some of the secondary outcome measures where participants that have withdrawn/lost-to-follow-up may potentially bias results. In addition to this, sensitivity analysis will be run on outcomes where protocol deviations may occur. These include investigations regarding missing data, and for the early restart of ART. Further details are provided within the SAP.

### Methods in analysis to handle protocol non-adherence and any statistical methods to handle missing data {20c}

There is a possibility that a small proportion of participants will be affected by COVID-19, an intercurrent event, and will need to restart ART before reaching the primary endpoint. This might affect the power, diluting the treatment effect. Other participants may also elect to restart ART before concluding follow-up without experiencing a viral rebound event. We expect this proportion to be very small and to stay within the 10% assumption used within the power calculation, but it will be monitored by trial oversight committees and sample size will be adjusted if needed. For these cases, under most scenarios censoring at time of ART restart will naturally handle missing outcome data. However, based on the pattern and extent of missingness, if appropriate, we will explore the effect of alternative missing data assumptions within sensitivity analysis using a multiple imputation approach. Further details of which will be included within the Statistical Analysis Plan.

### Plans to give access to the full protocol, participant level-data, and statistical code {31c}

Information concerning the study, patent applications, processes, scientific data, or other pertinent information is confidential and remains the property of the Sponsor. The investigator may use this information for the purposes of the study only.

The study protocol is published in this manuscript and will also be available via the clinicaltrials.gov registry. Statistical analysis plan and informed consent form will be available on request and subject to consent from the sponsor.

It is understood by the investigator that the Sponsor will use information developed in this clinical study in connection with the development of the IMPs and, therefore, may disclose it as required to other clinical investigators and to Regulatory Authorities. In order to allow the use of the information derived from this clinical study, the investigator understands that he/she has an obligation to provide complete test results and all data developed during this study to the Sponsor.

Verbal or written discussion of results prior to study completion and full reporting should only be undertaken with written consent from the Sponsor.

Therefore, all information obtained as a result of the study will be regarded as confidential, at least until appropriate analysis and review by the investigator(s) are completed.

Permission from the Trial Management Group is necessary prior to disclosing any information relative to this study outside of the Trial Steering Committee. Any request by site investigators or other collaborators to access the study dataset must be formally reviewed by the TSC.

A Clinical Study Report summarising the study results will be prepared and submitted to the REC within a year of the end of study. The results will also be submitted to the EudraCT results database in accordance with regulatory requirements.

#### Sub-studies

Investigators planning sub-studies may submit their proposals for consideration by the TSC, IDMC, and TMG. Planned or current sub-studies within the RIO trial includes a qualitative sub-study investigating the participant experience within the RIO study, and a sub-study for the collection of rectal biopsies in RIO participants, to investigate the pharmacokinetics of bNAbs and their impact on the gut HIV reservoir and mucosal immunity.

## Oversight and monitoring

### Composition of the coordinating centre and trial steering committee {5d}

A Trial Steering Committee (TSC) will be convened including as a minimum an independent Chair, independent clinician, the CI, and Trial Manager. The TSC charter is included in Supplement file 2. The role of the TSC is to provide overall supervision of trial conduct and progress. The TSC will meet approximately 6-monthly throughout the duration of the trial. Details of membership, responsibilities, and frequency of meetings will be defined in a separate Charter.

A Trial Management Group (TMG) will be convened including the CI, co-investigators and key collaborators, community representative, trial statistician, and trial manager. The TMG will be responsible for day-to-day conduct of the trial and operational issues. Details of membership, responsibilities, and frequency of meetings will be defined in separate Terms of Reference.

### Composition of the data monitoring committee, its role and reporting structure {21a}

A fully IDMC will be set up to monitor progress, participant safety, and any ethical issues involved in this trial. They will review trial progress, recruitment rates, and safety data. A separate IDMC Charter will be drawn up defining their responsibilities, frequency of meetings, and reporting to the TSC (Supplement file 3). Meetings will be approximately 6-monthly.

The statistician will analyse interim data for IDMC meetings and act as data manager, in raising and resolving data queries with participating sites, via the Trial Manager. Closed IDMC reports will include recruitment, randomisation balance and stratification effectiveness, baseline characteristics, unblinding, withdrawals, compliance, concomitant medications, efficacy, mediators, and adverse events. Open IDMC and TSC reports will be provided without outcome or arm information.

There are no statistical criteria (stopping rules) defined for termination of the RIO trial. The IDMC Charter will define procedures for early termination of the study due to safety, should this be required.

#### Endpoint Adjudication Committee

An independent panel of experts will be convened to review all viral rebound reports and confirm that these meet the trial definition for viral rebound. Copies of the viral rebound reports will be provided to the panel, along with redacted copies of any applicable lab reports and the outcome of the review will be recorded on the eCRF. The panel will agree their terms of reference at the start of the trial and will meet on a 6-monthly basis, or as required over the course of the trial.

### Adverse event reporting and harms {22}

An adverse event (AE) is defined as any untoward medical occurrence in a participant administered a medicinal product which does not have a causal relationship with the treatment. Serious adverse events (SAEs) are defined as any event that

- Results in death,
- Is life-threatening,
- Requires hospitalisation or prolongation of existing inpatient’s hospitalisation,
- Results in persistent or significant disability or incapacity,
- Is a congenital abnormality or birth defect.
- Important adverse events or reactions that do not meet the above criteria but may jeopardise a participant or require intervention to prevent one of the other outcomes above.

SAEs will be recorded throughout the study. All AEs, whether expected or not, will be recorded in the adverse event section of the case record form within 1 month of the form being due. Reporting of AEs will be coded using MedDRA hierarchy. Rapid reporting of all SAEs (within 24 h) must be performed, and the trial coordination centre will report all SAEs to the sponsor within 24 h. SAEs will be reviewed by the CI or a designated medically qualified representative to confirm expectedness and causality, within a reasonable timeframe as described in the study-specific safety reporting instructions. Reporting of SAEs will be via the trial data collection system, and copies of SAE reports sent to the sponsor throughout the trial. All AEs and SAEs reported by participating sites will be included in the trial publication

Decisions on management of AEs will be undertaken by the trial physician, such as referral to GP, hospital, or another clinic. The trial physician will also make decisions on the assignment of causality. If any doubt about the causality, the local investigator should inform the trial coordination centre who will notify the CI. For the purposes of the study, AEs will be followed up according to local practice until the event has stabilised or resolved, or the final follow-up visit, whichever is sooner.

All clinically important abnormal laboratory test results occurring during the study will be recorded as adverse events. These tests will be repeated at appropriate intervals until they return to baseline or a level deemed acceptable by the investigator and clinical monitor, or until a diagnosis that explains them is made.

Viral rebound will not be reported as adverse events as these are trial end points.

Immunological symptoms listed below are possible with administration of a monoclonal antibody and will be considered AEs of interest. Potential allergic-type reactions during and immediately following the administration of 10-1074-LS and 3BNC117-LS will be carefully monitored.

- Constitutional symptoms, e.g. fever, change in blood pressure, rigours/chills.
- Injection site reactions/extravasation changes, pruritis, urticaria, erythema, desquamation, ulceration.
- Serum sickness like syndromes as evidenced by fever, rash, arthralgia, arthritis, nephritis.
- Deposition of immune complexes in the kidneys leading to renal insufficiency.
- Anaphylaxis; adult respiratory distress syndrome, bronchospasm, or wheezing
- Cytokine release syndrome

Suspected unexpected serious adverse reactions (SUSARs) are defined as a SAR judged related to any dose of study drug administered, and not consistent with the applicable to the product information as set out in the IB for the investigational medicinal products. SUSARs should be notified to the appropriate regulatory authority, relevant REC, and the sponsor in accordance with regulatory requirements. SUSARs which are fatal or life-threatening will be reported not later than 7 days after becoming aware of the SAE. Any additional relevant information will be sent within 8 days of the report. Other non-fatal or life-threatening SUSAR will be reported within 15 days. Follow-up of participants who have experienced a SUSAR should continue until recovery is complete or the condition has stabilised. SUSAR reports should be unblinded prior to submission if required by national regulatory requirements.

Annual safety reports will be submitted to the sponsor, the ethics committee, and regulatory authority in accordance with regulatory requirements.

If a participant or their partner becomes pregnant while taking part in the trial where the foetus could have been exposed to an IMP, the investigator must ensure the participant and their healthcare professional are aware that follow-up information is reported on the outcome of the pregnancy. Each pregnancy will be reported to the ICTU within 24 h of learning of its occurrence. Any SAE experienced during the pregnancy and unrelated to the pregnancy must be reported on a SAE form. The pregnancy should be followed up to determine outcome, including spontaneous or voluntary termination, details of the birth, and the presence or absence of any birth defects, congenital abnormalities, or maternal and/or newborn complications. Pregnancy follow-up should be recorded on the same form and should include an assessment of the possible relationship to the investigational treatment.

If any urgent safety measures are taken, the CI/sponsor shall immediately and no later than 3 days from the date the measures are taken, give written notice to the MHRA and relevant REC of the measures taken and the circumstances giving rise to those measures.

### Frequency and plans for auditing trial conduct {23}

The study will be monitored periodically by trial monitors to assess the progress of the study, verify adherence to the protocol, GCP guidelines, and other national/international requirements and to review the completeness, accuracy, and consistency of the data.

The trial will require two separate monitoring roles:

(a) Blinded trial monitor responsible for reviewing informed consent, trial data (comparison of source data against eCRF), review and oversight of laboratory samples

(b) Unblinded trial monitor responsible for review of IMP supply and pharmacy aspects

A study-specific risk assessment will be performed prior to the start of the study to assign a risk category of ‘low’, ‘medium’, or ‘high’ to the trial. Risk assessment will be carried out by the ICTU Quality Assurance Manager in collaboration with the Study Manager and the result will be used to guide the monitoring plan. The risk assessment will consider all aspects of the study and will be updated as required during the course of the study.

Monitoring procedures and requirements will be documented in a monitoring plan, developed in accordance with the risk assessment.

Quality control will be performed according to ICTU internal procedures. The trial may be audited by a Quality Assurance representative of the Sponsor and/or ICTU. All necessary data and documents will be made available for inspection.

The study may be subject to inspection and audit by regulatory bodies to ensure adherence to GCP and the NHS Research Governance Framework for Health and Social Care (2nd Edition).

### Plans for communicating important protocol amendments to relevant parties (e.g. trial participants, ethical committees) {25}

Prior to the shipment of IMP and the enrolment of participant, the REC must provide written approval of the following: the conduct of the study at named sites, the protocol and any amendments, the Participant Information Sheet and Consent Form, any other written information that will be provided to the participants, any advertisements that will be used, and details of any participant compensation.

Proposed amendments to the protocol and aforementioned documents must be submitted to the REC for approval as instructed by the Sponsor. Amendments requiring REC approval may be implemented only after a copy of the REC’s approval letter has been obtained.

Amendments that are intended to eliminate an apparent immediate hazard to participants may be implemented prior to receiving Sponsor or REC approval. However, in this case, approval must be obtained as soon as possible after implementation.

The trial team, in collaboration with the Sponsor, will assess whether a proposed amendment is substantial or non-substantial. For each proposed amendment, a revised version of the protocol will be prepared using tracked changes, a new version number assigned, and the revised document will be reviewed and approved by Protocol Development Group and Sponsor prior to submission to the REC and Health Research Authority (HRA). The amended protocol will be sent to participating sites for local approval to be granted and the approved version will be shared with all staff involved in the trial.

### Dissemination plans {31a}

Trial findings will be shared with the community members of the TSC and TMG and advice sought on suitable dissemination strategies.

An annual meeting with all study participants and any chosen partner or family member will be held and updates on the research will be shared. The final study primary endpoint results will be first shared with the study participants prior to any public presentation of the findings in keeping with conference embargo rules. We will work the RIO trial community advisors to develop dissemination materials for public sharing of trial design, data, and main outcomes. Following this process, results will be peer-reviewed and published in international medical journals, conferences, and HIV information websites such as https://i-base.info.

## Discussion

### Role of community involvement

A cure for HIV has always been an ultimate community and scientific global goal. This is generally defined as being able to live a healthy disease-free life without relying on daily medication and also without risking HIV transmission to partners. There are multiple personal, global, implementation, and structural barriers to lifelong adherence to ART for the almost 40 million people worldwide living with HIV. While this challenge is significant, the increase in cure-related research over the last 10 years also gives people hope. Indeed, the US Martin Delaney Collaboratories for HIV Cure Research is named in memory of a leading HIV activist [23]. The global need for a cure comes not only for the wish to not be dependent on medicines, but not to be dependent on the vagaries of international donation programmes, and their susceptibility to political change.

The scientific design of the RIO trial will allow rigorous evaluation of the safety, tolerability, and efficacy of a treatment of long-acting dual bNAbs against HIV envelope compared with placebo to maintain viral suppression after stopping ART. The extended length of follow-up allows identification and determination of any mechanisms that underpin the post-viral control that may be observed; whether control is mediated directly via neutralizing antibody presence or whether there is an antibody-induced functional vaccinal effect [5]. In Stage 2, part of the trial, time to viral rebound for each individual can compare the first and second ATI period with potential to explore the underlying mechanisms involved.

As it should be with all research, community representation has been involved at all stages of the trial design. This helps researchers to design studies with participants’ values at the forefront, encouraging enrollment and which will help answer the most relevant study question. In this study, community views were involved in developing the two-stage design that would allow all participants access to bNAbs.

They also helped with designing acceptable criteria for the ATI, which is not part of routine care. Involvement in drafting participant information and consent forms by clearly explaining the potential risks and benefits in easy-to-understand language will also hopefully have more informed participants. These documents in the RIO study aim for a Flesch Reading Ease score of 70 or higher.

Community surveys can also help inform study design. But while these often report a high level of interest in cure research, the issues can be complex. This includes the following:

- That the personal expectations of participants who hope to be cured is often not shared as a likely outcome by the researchers.
- That survey responses might also overestimate the real likelihood of joining a study.
- Or that access to ART can affect levels of interest in different countries [24–27].

The inclusion of community representations therefore strengthened our study design. It resulted in easier to understand participant information, with easier readability, including an animated video. It helped with changes linked to COVID-19 and also made it easier for the study to be more widely publicised.

We included separate community representatives on the Trial Steering Committee, the Trial Management Group, and the Independent Data Monitoring Committee (IDMC). The makes the research a closer collaboration between professional researchers and the communities that the study ultimately aims to benefit.

The RIO study was planned to start in early 2020, just as SARS-CoV-2 was developing into a pandemic, and the community representations were closely involved in the discussions for when to pause and eventually reopen the study.

### Potential risks of the study

#### Risk of the intervention (bNAbs)

The use of bNAb infusions is experimental with, at time of writing, an estimated 27 (including 6 HIV-positive individuals) and 43 (including 15 HIV-positive individuals) study participants having received 10-1074-LS and 3BNV117-LS respectively. In total, 40 participants (including 5 HIV-positive individuals) have received both antibodies simultaneously in studies that had stopped recruitment or completed [28]. Studies with either bNAbs are ongoing (Table 1) and more data will be available on an ongoing basis.

As with all antibody infusions, there is a risk of anaphylaxis to the protein, and close monitoring will be necessary within a Clinical Research Facility. This requires well-trained clinical staff, access to intensive medical support if necessary, and monitored review of the participant both before, during, and at least an hour after completion of the infusion. To date, there have been no reported cases of anaphylaxis to either of the bNAbs included in the study [M Caskey personal communication]. Mild local and systemic reactions may be observed, and participants will have a weekly medical review including regular health screening questions, for the first 8 weeks after receipt of the infusions, and subsequently twice monthly. Infusion of antibodies carries a potential risk of thromboembolism, and coagulation screening tests will be performed at screening. Individuals at increased risk of thromboembolic disease will not be eligible for inclusion in the study.

#### Risk of undertaking ATI

In the absence of a surrogate marker of HIV cure, the only measure of post-treatment viral control is to interrupt antiretroviral therapy [29]. There are two key safety issues to consider when ART is discontinued. The first is the safety of the individual participant, including risk of HIV disease progression: CD4 decline, seroconversion symptoms with rapid viraemic rebound, potential development of ART drug resistance, and increase in size of the HIV reservoir upon viral rebound. However, recent evidence by Huiting et al [30] ATI causes neither expansion of HIV reservoirs nor immunological abnormalities following reinitiation of ART in individuals who initiated ART early. The international ATI consensus statement recommends participants included in ATI studies should have a functional immune system, likely to tolerate a period of viraemia, should have stable CD4 counts, and avoid inclusion of those with substantial or serious comorbidities including malignancies, and previous AIDS-defining illnesses [13]. The second risk is the risk of onward viral transmission to HIV-negative partners when the participants no longer have an undetectable viral load. In this trial, participants and their partners will be counselled on the risk of HIV transmission, and they will have access to effective HIV prevention strategies such as PrEP and condoms access.

#### Risks of ATI in the context of the ongoing COVID-19 pandemic

There is limited data on the impact of HIV on both SARS-CoV-2 acquisition; recent evidence suggests that people living with HIV may have worse outcomes and mortality of severe COVID-19 [31, 32]. It is unclear if this is driven by HIV or disparities in comorbidities and other confounding factors [33], or cellular immune deficiencies, as a previous study reported a low current or nadir CD4 being associated with worse outcomes [34]. In the context of ATI, the risk remains uncertain, but there is a hypothetical increased risk of severe COVID-19 outcomes for individuals with high HIV viral loads, and the associated immune activation and inflammation, or drop in CD4 T cell counts.

During the trial, all participants enrolled would have received at least one dose of the SARS-CoV-2 vaccine under the national vaccination programme. They would also be offered PPE and SARS-CoV-2 screening measures as described above, to mitigate the risk of SARS-CoV-2 acquisition during the trial.

### Benefits of the study

This randomised phase II study will determine whether a combination of dual long-acting bNAb may result in sustained HIV suppression in the absence of ART amongst individuals with immune recovery treated since primary HIV infection.

The study design allows the testing of two hypotheses, the first being that combination long-acting bNAbs will result in a sustained and quantifiable period of viral control following ATI compared with placebo amongst a specifically selected group of individuals who started ART in primary HIV infection.

The second hypothesis that bNAbs may drive sustained HIV control beyond the duration of detectable bNAb concentrations, through processes mediated by the host innate immunity. This is based on evidence that HIV may be controlled in the absence of antiretroviral therapy, in individuals often termed ‘elite controllers’, and this control is mediated by highly potent HIV-1-specific CD8 T cells [5]. In macaque SHIV models, depletion of CD8 T cells resulted in loss of viral control in monkeys who were virally suppressed following 3BNC117 infusions, suggesting CD8 T cells are responsible for control of virus replication in controller macaques. This process is possibly driven by formation of immune complexes from low levels of progeny virions prior to viral rebound, compared to the near complete inhibition of virus replication by ART limiting the amount of viral antigen to induce immunity [35]. It is unclear if similar CD8 T cell responses in humans may be elicited by bNAbs, and the role of ATI for providing the initial antigen production to drive this response [4]. This two-stage study design allows the investigation of this hypothesis, providing significant virological and immunological insights into bNAb-mediated viral control mechanisms that determine HIV control and remission. Early and continued community involvement throughout the study design and management will encourage stronger participant satisfaction and person-centred outcomes for the community of people living with HIV.

## Trial status

At the time of writing, the first participant was consented in May 2021 and recruitment is expected to complete in 2022. The current version of the protocol is 4.0, dated 5th March 2021.

### Supplementary Information

**Additional file 1.**

**Additional file 2.**

**Additional file 3.**

**Additional file 4.**

## Appendix 1

List of participating clinical sites

1. Imperial College Healthcare NHS Trust, London, UK

2. Guy’s and St Thomas Hospital NHS Foundation Trust, London, UK

3. Central and North West London NHS Foundation Trust, London, UK

4. Royal Free London Hospital NHS Foundation Trust, London, UK

5. Chelsea and Westminster Hospital NHS Foundation Trust, London, UK

6. Barts Health NHS Trust, London, UK

7. Brighton and Sussex University Hospitals NHS Trust, Brighton, UK

## Abbreviations

ACD: Acid-citrate-dextrose
ADA: Anti-drug antibodies
ADCC: Antibody-dependent cell-mediated cytotoxicity
AE: Adverse event
ALT: Alanine transaminase
ALP: Alkaline phosphatase
ART: Antiretroviral therapy
AST: Aspartate transaminase
ATI: Analytical treatment interruption
bNAbs: Broadly neutralising antibodies
bPI: Boosted Protease inhibitor
CI: Chief investigator
COVID-19: Coronavirus Disease 2019
DNA: Deoxyribonucleic acid
EDC: Electronic data capture
FBC: Full blood count
GCP: Good Clinical Practice
HBV: Hepatitis B virus
HCV: Hepatitis C virus
HIV: Human immunodeficiency virus
HTLV: Human T-lymphotropic virus
IB: Investigator brochure
ICRF: Imperial Clinical Research Facility
ICTU: Imperial Clinical Trials Unit
IDMC: Independent Data Monitoring Committee
IMP: Investigational medicinal product
INSTI: Integrase inhibitor
INR: International normalised ratio
LFT: Liver function test
LRA: Latency reversal agent
mAb: Monoclonal antibody
NGS: Next-generation sequencing
NHS: National Health Service
NHSE: National Health Service England
NIHR: National Institute for Health Research
MHRA: Medicines and Healthcare products Regulatory Agency
NK cell: Natural Killer cell
NNRTI: Non-nucleoside reverse transcriptase inhibitor
NRTI: Nucleoside reverse transcriptase inhibitor
PBMC: Peripheral blood mononuclear cells
PHI: Primary HIV infection
PI: Principal investigator
POCT: Point of care test
PEP: Post-exposure prophylaxis
PPE: Personal protective equipment
PrEP: Pre-exposure prophylaxis
RITA: Recent infection testing algorithm
REC: Research Ethics Committee
RNA: Ribonucleic acid
RPR: Rapid plasma reagin
RT-PCR: Real-time polymerase chain reaction
SAE: Serious adverse event
SAR: Serious adverse reaction
SGA: Single genome amplification
SOP: Standard operating procedures
SUSAR: Suspected unexpected serious adverse reactions
TSC: Trial steering committee
TMG: Trial management group
uPCR: urine protein creatinine ratio
VL: Viral load
