# Supplementary material for: The RIO trial: rationale, design, and the role of community involvement in a randomised placebo-controlled trial of antiretroviral therapy plus dual long-acting HIV-specific broadly neutralising antibodies (bNAbs) in participants diagnosed with recent HIV infection—study protocol for a two-stage randomised phase II trial
Source: Trials. 2022 Apr 5;23:263. doi: 10.1186/s13063-022-06151-w (PMC8981886; doi:10.1186/s13063-022-06151-w)
Supplement: Supplementary file 1 — Additional file 1. [file 13063_2022_6151_MOESM1_ESM.pdf]

Title: A randomised placebo controlled trial of ART plus dual long-acting HIV-specific broadly neutralising antibodies (bNAbs) vs ART plus placebo in treated Primary HIV Infection on viral control off ART

EudraCT: 2019-002129-31

Sponsor: Imperial College London

Funder: The Bill & Melinda Gates Foundation

MONITORING PLAN

RIO Version 1.0 01.05.2020

Prepared by	Title	Signature	Date
Ambreen Ashraf	Study Manager	Digitally signed by Ambreen Ashraf Date: 2020.05.06 04:40:36 +01'00'	
**Approved by**	**Title**	**Signature**	**Date**
Daphne Babalis	Operations Manager	Digitally signed by Daphne Babalis Date: 2020.05.06 08:41:14 +01'00'	
Ginny Picot	QA Manager	Digitally signed by Virginia Picot Date: 2020.05.06 16:36:09 +01'00'	

CONTENTS

1. Introduction: 3
2. Scope: 3
3. Abbreviations 4
4. Responsibilities 4
5. References 6
6. Risk Assessment: 7
7. Frequency and Extent of Monitoring: 7
8. Type of monitoring 8
9. Monitor Training 9
10. Monitor Site Capacity 10
11. Source Data Identifier document 10
12. Study Statistician Involvement 10
13. Guidelines for Monitoring Visits: 10
    13.1 Site Initiation Visit: 10
    13.2 Routine monitoring visit 11
        13.2.1 Prior to the Monitoring Visit 11
        13.2.2 During a Monitoring Visit: 12
        13.2.3 At the End of a Monitoring Visit: 14
        13.2.4 Monitoring Visit Report: 15
        13.2.5 Monitoring Visit Follow-Up Letter: 15
    13.3 Site Close Out Visit: 15
14. Identification of Data Errors and Non-Compliance: 16
15. Contact with Sites: 16
16. SAE review and reporting 17
17. Escalation 17
18. Revision History: 17

1. Introduction:

This Monitoring Plan has been developed to provide guidance on the preparation, conduct, reporting and follow-up of all study specific monitoring activities and requirements. The study-specific monitoring plan has been prepared in advance of trial initiation in accordance with the study risk-assessment (SOP QA004 Risk Analysis) and Site Monitoring (SOP CR015).

This study is a multicentre, placebo-controlled double-blinded two arm prospective phase II randomised controlled trial. This study will test the hypothesis that for individuals who commenced antiretroviral therapy (ART) in primary HIV infection (PHI), a combination of the two long-acting broadly neutralising antibodies, 3BNC117-LS and 10-1074-LS, will induce a period of virological remission when ART is stopped compared with participants who received ART plus placebo. The study is planned to run for five years from early 2020 and will recruit HIV-1 participants from mixed gender subjects ranging from ≥18 to ≤60 years of age across seven sites in the UK.

2. Scope:

This Trial Monitoring Plan describes the monitoring responsibilities of personnel involved in conducting the RIO Study.

The purpose of this Trial Monitoring Plan is to ensure that all trial monitoring undertaken as part of the RIO Study will follow a uniform set of instructions and monitoring tools that comply with the Monitor's responsibilities as set out in the study protocol and the latest revision of the principles of the "Declaration of Helsinki", Good Clinical Practice (ICH GCP E6(R2) Guidelines) and the UK Medicines for Human Use (Clinical Trials) Regulations.

3. Abbreviations

AE – Adverse Events
bNabs- Broadly Neutralising antibodies
COV – Close-Out Visit
ICTU - Imperial Clinical Trials Unit
IMP - Investigational Medicinal Product
ISF – Investigator Site File
MV – Monitoring Visit
MVR - Monitoring Visit Report
NHS – National Health Service
PSF – Pharmacy Site File
PI - Principal Investigator
QA – Quality Assurance
R&D – Research & Development
SAE – Serious Adverse Events
SDV - Source Data Verification
SIV - Site Initiation Visit
SOP – Standard Operating Procedure
SSPM – Study Specific Procedure Manual
TMF – Trial Master File
UK – United Kingdom

4. Responsibilities

**Chief Investigator**	• Have awareness of trial progress and provide support to the Trial Manager/Monitor, as necessary.<br>• Provide input into the study-specific Risk Assessment as detailed in SOP QA004.
**Operations Manager (blinded)**	• Oversee monitoring activities for the trial.<br>• Prepare the Monitoring Plan with the Trial Manager/Monitor<br>• Review and approve monitoring visit reports and follow-up letters if the Trial Manager is

	unavailable, or if the Trial Manager is responsible for monitoring.
**Operations Manager (unblinded)**	• Oversee unblinded monitoring activities for the trial<br>• Review and approve monitoring visit reports and follow-up letters if the Trial Manager is responsible for monitoring
**Study/Trial Manager (combined with blinded Trial Monitor role)**	• Prepare the Monitoring Plan, and any updated versions required during the course of the trial.<br>• Perform monitoring activities for the trial.<br>• Ensure the blinded Monitor is appropriately trained in the Monitoring Plan and applicable SOPs.<br>• Perform Site Initiation Visits, Interim monitoring visits and Close-Out Visits.<br>• Performing data checks on the study database throughout the study to ensure data quality and accuracy.
**Blinded Trial Monitor (combined with Trial manager role)**	• Prepare the Monitoring Plan.<br>• Prepare for, schedule and conduct monitoring activities (SIVs, MVs and COVs).<br>• Prepare monitoring visit reports and follow-up letters in a timely manner.<br>• Maintain training records to demonstrate appropriate level of knowledge and understanding of assigned trials.<br>• Perform data checks on the study database throughout the study to ensure data quality and accuracy.
**Unblinded Trial Monitor**	• Reviews the Monitoring Plan.<br>• Prepare for, schedule and conduct monitoring activities in relation to drug accountability.<br>• Create monitoring visit reports and follow-up letters in a timely manner.<br>• Maintain training records to demonstrate appropriate level of knowledge and understanding of assigned trials.<br>• Ensure blinding to patient IMP allocation is maintained at all times.

**Study Statistician**	• Contribute to data cleaning as required such as checking for any unusual patterns or trends in the study data.<br>• Provide a random generated list of participant IDs and visits for SDV where appropriate.
**QA Manager**	• Be involved in discussions regarding non-compliance and serious breaches.<br>• Ensure that the Monitoring Plan incorporates monitoring of the risks identified as part of the current risk assessment for the study.<br>• Review and approve the Monitoring Plan.<br>• Conduct the study Risk Assessment in collaboration with the study team to determine the study risk and associated level of study monitoring required.

5. References

1	International Conference on Harmonisation E6 Guideline for Good Clinical Practice
2	Integrated Addendum to ICH E6(R1): Guideline for Good Clinical Practice, E6 (R2), Step 5, Dated 1st December 2016
3	Risk-adapted Approaches to the Management of Clinical Trials of Investigational Medicinal Products, 2011, MRC, DH, and MHRA
4	JRCO SOP 015 – Monitoring Clinical Trials
5	SOP HR001 Staff Induction and Training
6	SOP CR012 Study Specific Procedure Manuals
7	SOP CR013 Initiation of Investigator Sites
9	SOP CR016 Safety Reporting for Clinical Trials of Investigational Medicinal Products (CTIMP) and Non-CTIMPs.

10	SOP CR017 Detection and Management of Non-Compliance at Investigational Sites
11	SOP CR015 Site Monitoring
12	SOP CR018 Site Closeout Visits
13	SOP CR022 Serious breaches of GCP and Protocol
14	SOP QA004 Risk Analysis
15	MHRA Good Clinical Practice Guide 2012
16	SOP GA11 Escalation Procedures

6. Risk Assessment:

A risk assessment of the study has been completed by the Study Team and QA Manager on 28th October 2019 placing this study as **MEDIUM** risk with respect to monitoring, based on protocol risk. Medium Risk areas identified were "Study Participants", "Validity of Study Results" and "Study Organisation". Responsibility risk elements identified for the RIO trial, which will be used to determine audit are; Central Ethics and Regulatory approvals, eCRF set up, Randomisation, Study Monitoring, SAE assessment and SUSAR reporting, Data Management and Analysis, Statistical Analysis and Brexit. As the antibodies used are unlicensed, RIO has been rated High risk overall. For further information, please refer to risk analysis document in the RIO TMF.

7. Frequency and Extent of Monitoring:

The MEDIUM risk classification of RIO trial requires the following monitoring schedule:

VISIT TYPE	FREQUENCY	MONITORING DETAILS / SDV
Site Initiation Visit	1 per site	Full RIO training; Investigator's Site File (ISF), Pharmacy file (Imperial site)
Routine Monitoring Visit	2 visits (or 1-3) per annum	• Source Data Verification (SDV) of 50% of subjects, existence, primary endpoints, AEs<br>• 100% SDV for first patient randomised at each site<br>• SDV of 100% of consent forms and SAEs<br>• Verification of research approvals, study protocol

		• Drug delivery and drug accountability by unblinded monitor (ICRF only)<br>• Patient Information documents and archiving arrangements<br>• Investigator Site File (ISF) at each site (will not be assessed at each visit) Pharmacy Site File (PSF) at ICRF by unblinded monitor.
Close-out visit	1 per site	Full InForm query review, archiving procedures; IMP destruction

Following the site initiation visit, the first interim monitoring visit at each site will take place within 1 month of the first patient being randomised into the study. This is to ensure patient safety, accurate data collection and reporting. If the first monitoring visit is not undertaken within the 2 months window, a file note should be prepared outlining the reason for the delay. Subsequent to the visit, interim monitoring visits will be conducted twice per year at each site. However, the first ICRF monitoring visit will take place within a month of the first patient receiving bNabs. Additional interim site monitoring visits may be performed due to high recruitment or where this is required due to performance, compliance or other issues.

Based on the frequency of monitoring visits required, a monitoring visit tracker SOP_TEM_CR022 version 1 will be prepared to log and track all monitoring activities performed at each site.

The intervals for monitoring visits may be discussed with the Trial Manager/Operations Manager and revised dependent on subject enrolment rate, quality issues, site compliance or other trial issues. Any significant deviation from the planned monitoring timelines will be explained and documented in the monitoring visit report and the plan amended if appropriate.

8. Type of monitoring

The following type of monitoring will be conducted during the course of trial and primarily on-site monitoring will be conducted. The frequency and extent of monitoring is described in section 7.

8.1 Central Data Monitoring

Central monitoring of data using statistical techniques or data reports for identification of unusual patterns of data and to detect sites or individuals where there may have been deviations from the protocol. Examples of central monitoring techniques which may be used include:

- Missing or invalid data (range checks)
- Calendar checks
- Unusual data patterns
- Repeated measures
- Protocol Deviations
- Confirmation of consented patients
- Ensuring that the Clinical Team are entering data in a timely manner
- Study dose assessed using eCRF
- Review and monitor AEs
- Review recruitment levels against targets
- Delegation of responsibilities and site signature log reviewed

8.2 On-site monitoring

On-site monitoring visits will be used to confirm patient eligibility, to corroborate the existence of the patient, review of critical data for primary outcome and to complete source data verification and review of patient safety as highlighted in section 7 above.

9. Monitor Training

Monitors are expected to acquire an appropriate level of knowledge and understanding of the following:

- RIO trial protocol
- Investigational Medicinal Product (IMP)
- Participant Information Sheet (PIS) Informed Consent Form (ICF), and any other relevant trial-specific documents.
- Monitoring requirements of assigned trials (as detailed in the Monitoring Plan).
- All RIO study specific Study Specific Procedure Manuals (SSPM).
- Applicable regulatory requirements

Training in these areas will have been completed and documented (Certification of training, SOP_TEM_CR010, formerly CR012D-T) before monitoring of a trial is initiated. It is the Monitor's responsibility to ensure that their training records are kept up-to-date (See SOP

GA002). Monitor/s will ensure that they update their training records (including SOP training, GCP training and study specific training) whenever new training or SOP notifications has been provided.

10. Monitor Site Capacity

There will be **two** Trial Monitors assigned to RIO trial – **Blinded** Trial Monitor (combined with Trial Manager role) and an **Unblinded** Trial Monitor. Blinded Monitor will be responsible for the majority of on-site monitoring activities (as specified in section 13) with the exception of drug delivery and accountability. The Unblinded trial monitor will be responsible for the monitoring of drug accountability including IMP storage, handling procedures and IMP orders/transportation, to the Imperial Clinical Research Facility (ICRF) located in Hammersmith Hospital. Monitoring will be conducted as described in section 8.0 above.

11. Source Data Identifier document

A Source Data Identifier document will be created by the Study Manager, with input from the Chief Investigator and the Operations Manager. This will state the various source documents that will be used by participating sites to collect individual data from, and which may be verified during source-data verification.

12. Study Statistician Involvement

The Monitor must provide the Study Statistician with ample time to generate a random list of *participant* IDs and visits for SDV during on-site monitoring visits.

At regular time points (as per the Data Management Plan), the Study Statistician will contribute to data cleaning by running statistical reports on the study data for inconsistencies and discrepancies, reporting back to the Study Manager/Monitor.

13. Guidelines for Monitoring Visits:

13.1 Site Initiation Visit:

The Site Initiation Visit (SIV) will take place as per SOP CR013, when there is assurance that the site agreement(s) and all relevant regulatory approvals are in place or otherwise agreed with the QA manager. This will be arranged so that the PI will be able to attend, alongside any other key members of the study team (e.g. Study Doctor, Research Nurse, Pharmacist (where applicable)). A list of attendees will be collected. A presentation will be given to the site and will include at least the following:

- Study background
- Aims/outcomes of the trial
- Inclusion/exclusion criteria
- Study Visits and procedures
- Trial Documentation
- GCP and staff training
- Informed consent documentation
- Safety Reporting
- Study timelines
- Data collection and eCRF (including source data expectations)
- Monitoring and auditing procedures
- Access to source data, including electronic source records, for the purpose of SDV
- Protocol deviations and violations
- Archiving
- ISF maintenance
- Amendments
- Contact details
- IMP storage and dispensing
- Pharmacy procedures
- PSF maintenance
- Laboratory (where applicable), and equipment calibration

A SIV report will be completed after the visit to record the topics covered and any issues raised (see SOP_FRM_CR018, formerly CR013C-F). The report will be approved by the Operations manager (or delegate), and a signed, finalised paper copy filed in the ISF and TMF.

13.2 Routine monitoring visit

13.2.1 Prior to the Monitoring Visit

Each visit will be arranged at a mutually convenient date and time between the site staff and Study Monitor, either by email or telephone. A formal confirmation letter or email confirming the arrangements of the monitoring visit, will be created by the Study Monitor. (see SOP_TEM_CR016, formerly CR015B-T Monitoring Visit Confirmation Letter template). This will be sent to the Principal Investigator (PI) and site research team for filing in the Investigator Site File (ISF). A copy will also be filed in the TMF.

The Study Monitor/Manager will prepare for these visits by undertaking the following activities:

- Review recruitment rates at the site.
- Review the ISF in order to ascertain which documents must be collected from the site and filed in the TMF.
- Review the InForm/ Study database data for unresolved queries and outstanding data entry.
- Review any AE's and SAE's reported at the site.
- Prepare an enrolment list (or generate a report from InForm where possible) to confirm which subjects were randomised, so that this can be checked against IMP accountability records at site to ensure correct dispensing.
- Identify ancillary departments to be visited e.g. pharmacy, labs.

The Study Monitor will also review the last monitoring visit report, recent telephone contact report(s) and/or emails with the site staff and any other relevant study updates (including outstanding actions) to establish the monitoring activities to be performed during the scheduled visit. The Study Monitor (blinded) will also liaise with Study Manager/blinded monitor and Study Statistician to see if there are any outstanding issues with the site from their perspective.

13.2.2 During a Monitoring Visit:

During the monitoring visit, the Study Monitor will perform Source Data Verification (SDV), on participants identified by the trial statistician. The Monitor will take into account the risks identified in the risk-assessment and focus on these aspects during monitoring visits.

**MONITORING ACTIVITIES FOR BLINDED TRIAL MONITOR**
Review Participant Informed consent forms (original version and any amendments as applicable)
Complete Source Data Verification (SDV) as per requirements of the risk assessment.<br>Review lab/medical reports to ensure Investigator (or designee) review and sign off on all lab reports
Review data quality<br>• Ensure data has been entered on the trial database accurately, compared to the source<br>• Ensure any calculations have been done correctly<br>• Ensure missing or outstanding data is entered as soon as possible<br>• Verify source data for reported and any unreported adverse events

• Verify source data for reported and any unreported protocol deviations/violations<br>• Assist the site to resolve data queries
Review the Investigator Site File<br>• File/collect essential documents (e.g. CVs of new staff, updated insurance certificates/IB etc.).<br>• Ensure the SAE tracking log is up to date (generated from InForm where possible) and filed in the Investigator Site File<br>• Ensure that all R&D reporting requirements have been met as per SOPs CR007, CR016, CR020, CR021 (e.g. Reporting of SAEs to R&D as per NHS Permission/R&D Approval, approval for substantial amendments)<br>• Review and take copies of updated Logs for TMF;<br>e.g. Delegation of Duties and Site Signature Log<br>• Ensure trial logs have been updated<br>o Screening/Randomisation Log.<br>o Participant Identification Log (not to be copied for Trial Master File at ICTU as this contains participant identifying information)<br>o Protocol Deviation/Violation logs<br>o Sample/tissue collection logs etc.<br>o IMP/treatment accountability logs (if not in Pharmacy)<br>o Sign the Monitoring Visit Log
Discussion with site staff including Principal Investigator on:<br>• New issues and unresolved issues from previous monitoring visit<br>• Patient recruitment<br>• Reminder on SAE reporting requirements and to check status of SAE (resolved, ongoing, stop date etc.)<br>• Sites compliance to protocol<br>• Timing of next visit
Laboratory/Biological Samples<br>• Verify labelling and storage of biological samples, including temperature controls<br>• Ensure that logs for sample processing, storage and shipment are being maintained<br>• Ensure laboratory normal reference ranges and accreditations are up-to-date<br>• Ensure that samples are only being sent to the contracted laboratory<br>The above must be performed in accordance with the RIO study-specific manuals.<br><br>Central Laboratory Monitoring<br>• Verify laboratory staff have conducted laboratory specific GCP training

• Ensure laboratory staff processing research samples have protocol and study specific training proportionate to their role in sample processing such as sample labelling/identification, reporting of results, adaptation of a specific assay and storage of the sample/data<br>• Ensure that a Central Laboratory File and training records are maintained such as CV, recent GCP training, job description, assay competency assessments and protocol specific training.<br>• Perform on-site visit to central laboratories (at least one visit over the course of the study) to verify storage conditions and compliance with study procedures and regulatory requirements
**MONITORING ACTIVITIES FOR UNBLINDED TRIAL MONITOR**
IMP accountability:<br>• Review storage conditions of the IMP including review of temperature log, and immediate reporting to Study Manager of any excursions<br>• Reconcile the IMP supply used (empty IMP vials) and IMP available at the pharmacy against the inventory and subject accountability logs<br>• IMP vials will be destroyed immediately to help protect the blinding process<br>• Review randomisation lists and IMP allocations<br>• Review any new IMP shipment documentation<br>• Review of the relevant section of the ISF for IMP management/Pharmacy folder<br>• Prepare accountability reports and store separately from the TMF to ensure blinding

13.2.3 At the End of a Monitoring Visit:

At the end of the monitoring visit, the Study Monitor will arrange to meet with the PI and/or other relevant site staff to discuss the findings of the visit and to outline any action points to be resolved. This will give the site an opportunity to respond to any queries and suggest corrective action(s). The PI is expected to be available for an hour at the end of every monitoring visit. If this is not possible, a phone call or email follow up should be conducted with the PI after the visit and this should be documented in the monitoring report. The next monitoring visit date may also be arranged. A monitoring visit log will be completed by the Study Monitor, which will be counter-signed by the site staff (see CR015C-F Monitoring Log). The original log should be maintained in the ISF and a copy collected for the TMF, at the end of the trial.

13.2.4 Monitoring Visit Report:

Following a monitoring visit, the Study Monitor will prepare a Monitoring Visit Report (MVR) using the template (see SOP_TEM_CR017, formerly CR0154D-F). The MVR will be approved by the Operations Manager (or delegate). The original signed, finalised paper copy of the report will be filed in the TMF within 3 weeks of the monitoring visit.

The unblinded trial monitor will prepare a separate Unblinded-Monitoring Visit Report (based on the same template), which will be filed separately at the coordinating centre, so that it is only accessible by the unblinded monitor. Unblinded report will be reviewed by unblinded Operations Manager.

13.2.5 Monitoring Visit Follow-Up Letter:

A monitoring visit follow-up letter will be created by the Study Monitor using the follow up letter template (see SOP_TEM_CR018, formerly CR015E-T Monitoring Visit Follow-Up Letter), to discuss the monitoring activities completed, the findings and action points resulting from the visit. The letter will be sent to the PI not later than 1 week after the MVR is signed off. A signed, finalised copy of the letter may also be sent to the site by email or post, for filing in the ISF. A signed paper copy will be filed in the TMF.

A separate Monitoring Follow-Up Letter (using the same template, SOP_TEM_CR018) will be prepared by the unblinded Trial Monitor to discuss the monitoring activities completed, the findings and action points resulting from the visit. The letter will be sent to the unblinded research personnel, including the site pharmacy, not later than 1 week after the MVR sign-off. This letter should be clearly marked as Confidential for Unblinded RIO research staff only. It should be filed in the Site Pharmacy File.

13.3 Site Close Out Visit:

A site Close Out Visit (COV) will be performed at each site after the trial has been completed. (or, if the site has been asked to stop recruitment prematurely, a site closeout visit will be performed after the final follow-up visit). A remote close out is an option for the sites with no recruitment (if applicable). Prior to the visit the Sponsor must be satisfied that all data has been received and entered onto the CRF/ eCRF and that there are no outstanding queries. The Study Monitor will arrange the visit at a convenient date and time with the site staff and will notify them in writing. Site close out procedures will be performed in accordance with the ICTU SOP Site Closeout Visits (CR018).

Pharmacy department may be closed out separately to the main site for studies with a long follow-up period in which case the pharmacy file. The pharmacy file should be retained and the pharmacy department until all participants visits are completed or all patients unblinded (whichever occurs sooner), to ensure the research team are not unblinded.

A site COV report will be completed within 2-3 weeks of the visit to record the topics covered and any issues raised (see SOP_FRM_CR025, formerly CR018A-F, Site Close-out Monitoring Visit Report). The report must be approved by the Operations Manager (or delegate) and a signed, finalised paper copy will be filed in the TMF. This report is to be completed if a joint monitoring visit/COV is to be performed.

The Study Monitor/ Study Manager will write a follow-up letter to the site summarising the close out activities performed and identify any issues raised. The signed, finalised letter can be sent to the site by email or post. A signed paper copy will be filed in the TMF.

14. Identification of Data Errors and Non-Compliance:

The Blinded Study Monitor will discuss any discrepancies in source data or protocol non-compliance identified during the monitoring visits (or during communication with the investigational sites otherwise) with the PI and the Operations Manager upon notification. The Unblinded Study Monitor will discuss any discrepancies with the unblinded research personnel and the unblinded Operations Manager before he or she discuss it with the Trial Manager and the PI. Monitor must ensure not to unblind study manager and the PI. Any urgent issues should be discussed with the Trial manager without disclosing the site number/name. A discussion of the findings, together with any corrective action(s) will be documented in the monitoring visit report.

For more details and definitions of protocol deviations/violations and how to manage these please refer to SOP CR017 Detection and Management of Non-Compliance at Investigational Sites.

15. Contact with Sites:

Contact should be maintained by the Study Monitor between site visits by telephone, fax or email. Teleconferences can be held between site staff and the Study Monitor (or delegate) if important issues need to be discussed (i.e. actions to be resolved on the MVR). A signed copy of the contact report form (see SOP_FRM_CR021, formerly CR015 04F-F Contact Report Form) will be filed in the TMF.

16. SAE review and reporting

Monitors will review 100% SAEs as part of the SDV process but must also remind site staff at each monitoring visit to report all SAEs within 24 hours of being aware of the event, and to follow-up the outcome status of the SAE until it is resolved. Failure to adhere to the SAE reporting timelines must be reported as a protocol violation. Repetitive non-compliance with reporting of SAEs could be reported to the regulatory authorities as a 'serious breach'.

17. Escalation

Issues of non-compliance should be managed as per section 14. Should resolution not be achieved then the non-compliance will be escalated as per SOP GA11.03.

18. Revision History:

Version	Date	Reason for Update
1.0	01.05.2020	Final version
