# Supplementary material for: The RIO trial: rationale, design, and the role of community involvement in a randomised placebo-controlled trial of antiretroviral therapy plus dual long-acting HIV-specific broadly neutralising antibodies (bNAbs) in participants diagnosed with recent HIV infection—study protocol for a two-stage randomised phase II trial
Source: Trials. 2022 Apr 5;23:263. doi: 10.1186/s13063-022-06151-w (PMC8981886; doi:10.1186/s13063-022-06151-w)
Supplement: Supplementary file 2 — Additional file 2. [file 13063_2022_6151_MOESM2_ESM.pdf]

The RIO Trial: A randomised placebo controlled trial of ART plus dual long-acting HIV-specific broadly neutralising antibodies (bNAbs) vs ART plus placebo in treated Primary HIV Infection on viral control off ART

EudraCT: 2019-002129-31

Sponsor: Imperial College London

Funder: Bill and Melinda Gates Foundation

Trial Steering Committee Charter

Version 1.0, 06/01/2020

**Prepared by:**<br>**Name:**	**Title:**	**Signature:**	**Date:**
Hanna Box	RIO Project Manager	Hanna Box Digitally signed by Hanna Box DN: cn=Hanna Box, o=Imperial College London, ou=Department of Infectious Disease, email=h.box@imperial.ac.uk, c=GB Date: 2020.01.30 12:29:06 Z	**30.01.2020**
**Approved by:**<br>**Name:**	**Title:**	**Signature:**	**Date:**
Daphne Babalis	Operations Manager	Daphne Babalis Digitally signed by Daphne Babalis Date: 2020.01.30 12:36:18 Z	
**Approved by:**<br>**Name:**	**Title:**	**Signature:**	**Date:**
Frank Post	TSC Chair		

Contents

CONTENTS 2

ABBREVIATIONS 3

1. PURPOSE OF DOCUMENT 4

2. ROLE AND RESPONSIBILITIES 4

3. MEMBERSHIP 4

3.1. TSC Members 5

4. MEETINGS 5

4.1. Schedule and Process 5
4.2. Voting Rules 6
4.3. Meeting Minutes 6
4.4. Recommendations 6

5. REVISION HISTORY 6

APPENDIX 1: RELATIONSHIP OF TRIAL COMMITTEES 7

APPENDIX 2: AGREEMENT AND POTENTIAL COMPETING INTERESTS FORM 8

Abbreviations

ART	Antiretroviral Therapy
BNAB	Broadly Neutralising Antibody
CI	Chief Investigator
EUDRACT	European Union Drug Regulatory Agency Clinical Trial
GCP	Good Clinical Practice
ICTU	Imperial Clinical Trials Unit
IDMC	Independent Data Monitoring Committee
TMG	Trial Management Group
TSC	Trial Steering Committee

1. Purpose of Document

The purpose of this document is:

- To define the responsibilities of the Trial Steering Committee (TSC)
- To identify the TSC members
- To document the information required by the TSC
- To provide a communication plan

2. Role and Responsibilities

The primary role of a TSC is to provide overall supervision of the trial and ensure that it is being conducted in accordance with the principles of Good Clinical Practice (GCP) and the relevant regulations. The TSC should agree the trial protocol and any protocol amendments and provide advice to the investigators and the Trial Management Group (TMG), via Imperial Clinical Trials Unit (ICTU) on all aspects of the trial.

In practice these responsibilities are carried out by performing the following functions:

- Ensure that the rights, safety and well-being of the trial participants are the most important considerations and should prevail over other interests.
- Monitoring and review of the trial at regular TSC meetings to ensure the project is progressing according to a suitable time-scale and that deadlines defined in the protocol for aspects such as recruitment, data collection and compliance are being met.
- Consider new information from the TMG and Independent Data Monitoring Committee (IDMC), via ICTU and recommend appropriate action such as changes to the protocol, addition of patient information, or stopping or extending the study to ICTU.
- Provide advice, through its chair, to ICTU / Chief Investigator (CI), on all appropriate aspects of the trial. ICTU / CI will pass on this information to the Sponsor and Trial Funder.

The relationship with other Trial Committees is depicted in Appendix 1.

3. Membership

TSC membership is limited to:

- Independent Chair (with experience in clinical trials)
- Independent Clinician(s), Statistician and any others (e.g. health economist) with expertise relevant to the project
- Community Representative
- Chief Investigator, Co-investigators
- Representatives of the trial team who will attend meetings: Trial Manager, Project Manager, Study Statistician (and Senior Statistician if required) and Operations Manager

The Sponsor or funder may request to send official letters of invitation to members of the TSC.
Representatives of the Trial Sponsor and the Trial Funder should be invited to all TSC meetings.

All members are asked to sign an Agreement and Potential Competing Interests Form (Appendix 2) and provide a copy of their CV to the Study Team via email to rio_trial@imperial.ac.uk.

3.1. TSC Members

Institution	Name	Role	Voting Rights
King's College London	Prof Frank Post	Chair, Clinician	Yes
University College London	Prof Caroline Sabin	Statistician	Yes
University of Edinburgh	Prof Clifford Leen	Clinician	Yes
University of Sussex	Dr Jaime Vera	Clinician	Yes
University of Oxford	Dr Dimitra Peppa	Clinician	Yes
UK-CAB	Mr Ben Cromarty	Community Representative	Yes
Imperial College London	Prof Sarah Fidler	Study CI, Clinician	Yes
University of Oxford	Prof John Frater	Protocol Co-Chair, Clinician	Yes
The Rockefeller University	Prof Michel Nussenzweig	Study Co-Investigator, Clinician	No

4. Meetings

4.1. Schedule and Process

The TSC should meet approximately every 6 months, although there may be periods when more frequent meetings are necessary.

Meetings should be organised by the Trial Manager. The members should attend meetings as appropriate, and participation in meetings can be via teleconference in order to minimise demand on time.

Where an IDMC is required, TSC meetings should be scheduled to follow shortly after the IDMC meetings so that recommendations from the IDMC can be considered.

Relevant documents for the meeting will be prepared and distributed in advance by Trial Manager. As a minimum these documents will include:

- Agenda
- Previous minutes

- Update on study progress to include information on the status of participating sites, recruitment, safety, updates from TMG and IDMC, upcoming analyses, protocol amendments and any other pertinent information.

If applicable, any tables and analyses specified in the interim analysis plan or statistical analysis plan will also be provided to the TSC.

4.2. Voting Rules

Voting rights of specific TSC members is detailed in Section 3.1.

Voting is by simple majority. To be considered quorate a minimum of 3 independent members should be present including the independent Chair.

4.3. Meeting Minutes

Accurate minutes will be prepared following each meeting by the Trial Manager and agreed by all members. The signed finalised minutes will be sent via email to each TSC member. Copies of the minutes will be filed in the Trial Master File.

4.4. Recommendations

The TSC will be asked to comment in detail on suggested substantial changes to the protocol, extension requests or any other recommendations.

5. Revision History

Version Number	Date Effective	Reason for update
0.1	28-Oct-2019	First draft version
1.0	6-Jan-2020	First approved version

Appendix 1: Relationship of Trial Committees

The diagram demonstrates the main trial committees, shows how all communications between committees should pass through ICTU (although not always through the same person) and (by use of colour) shows how ICTU is represented on TMG and TSC and how TMG is represented on TSC.

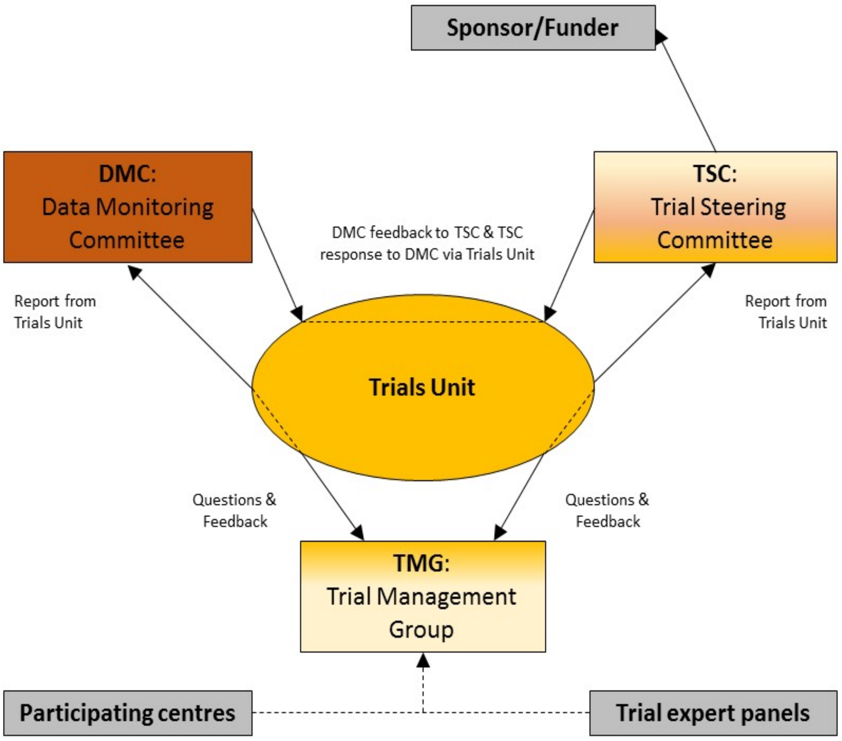

Appendix 2: Agreement and Potential Competing Interests Form

Agreement to join the RIO Trial Steering Committee (TSC) and disclosure of potential competing interests.

Please complete the following document and return to the Study Team by e-mail (rio_trial@imperial.ac.uk)

(**please initial box to agree**)

☐ I have read and understood the TSC Charter version 1.0, dated 06/01/2020

☐ I agree to join the TSC for this trial

☐ I agree to treat all sensitive trial data and discussions confidentially

The avoidance of any perception that members of a TSC may be biased in some fashion is important for the credibility of the decisions made by the TSC and for the integrity of the trial. Possible competing interest should be disclosed via email to the Study Team on rio_trial@imperial.ac.uk. In many cases simple disclosure up front should be sufficient. Otherwise, the (potential) TSC member should remove the conflict or stop participating in the TSC.

Table 1 lists potential competing interests.

(**please tick appropriate box**)

☐ No, I have no competing interests to declare

☐ Yes, I have competing interests to declare (please detail below)

Please provide details of any competing interests:

____

____

____

Name: ______________________________

Signed: ______________________________ Date: ________________

Table 1: Potential competing interests

• Stock ownership in any commercial companies involved<br>• Stock transaction in any commercial company involved (if previously holding stock)<br>• Consulting arrangements with the Sponsor<br>• Frequent speaking engagements on behalf of the intervention<br>• Career tied up in a product or technique assessed by trial<br>• Hands-on participation in the trial<br>• Involvement in the running of the trial<br>• Emotional involvement in the trial<br>• Intellectual conflict e.g. strong prior belief in the trial's experimental arm<br>• Involvement in regulatory issues relevant to the trial procedures<br>• Investment (financial or intellectual) or career tied up in competing products<br>• Involvement in the publication in the form of authorship
