# Supplementary material for: The RIO trial: rationale, design, and the role of community involvement in a randomised placebo-controlled trial of antiretroviral therapy plus dual long-acting HIV-specific broadly neutralising antibodies (bNAbs) in participants diagnosed with recent HIV infection—study protocol for a two-stage randomised phase II trial
Source: Trials. 2022 Apr 5;23:263. doi: 10.1186/s13063-022-06151-w (PMC8981886; doi:10.1186/s13063-022-06151-w)
Supplement: Supplementary file 3 — Additional file 3. [file 13063_2022_6151_MOESM3_ESM.pdf]

The RIO Trial: A randomised placebo controlled trial of ART plus dual long-acting HIV-specific broadly neutralising antibodies (bNAbs) vs ART plus placebo in treated Primary HIV Infection on viral control off ART

EudraCT: 2019-002129-31

Sponsor: Imperial College London
Funder: Bill and Melinda Gates Foundation

Independent Data Monitoring Committee Charter

Version 1.0, 06/01/2020

**Prepared by: Name:**	**Title:**	**Signature:**	**Date:**
Hanna Box	RIO Project Manager	Hanna Box [illegible]	**30.01.2020**
**Approved by: Name:**	**Title:**	**Signature:**	**Date:**
Daphne Babalis	Operations Manager	Daphne Babalis Digitally signed by Daphne Babalis Date: 2020.01.30 12:37:49 Z	
**Approved by: Name:**	**Title:**	**Signature:**	**Date:**
Abdel Babiker	IDMC Chair		18/2/2020

Note: This DMC charter was developed using the MRC Clinical Trials Unit template DMC Charter version 2.01, 13-Mar-2006

Contents

1 Introduction 4
2 Roles and Responsibilities 4
3 Before or early in the trial 5
4 Composition 6
5 Relationships 7
6 Organisation of meetings 7
7 Trial documentation and procedures to ensure confidentiality and proper communication 8
8 Decision making 10
9 Reporting 11
10 Release of results 11
11 Revision History 12
12 Appendix 1: Diagram summarising trial 13
13 Appendix 2: Agreement and potential competing interests form 15
14 Appendix 3: Agreement and confidentiality agreement for observers 16
15 Appendix 4: Relationship of Trial Committees 17
16 Appendix 5: Suggested report from IDMC to TSC where no recommendations are being made 18
17 Appendix 6: Guidelines from US bNAb trials Safety Committee 19

Abbreviations

AE	Adverse event
AR	Adverse reaction
ART	Antiretroviral Therapy
BNAB	Broadly Neutralising Antibody
CI	Chief Investigator
EUDRACT	European Union Drug Regulatory Agency Clinical Trial
GCP	Good Clinical Practice
ICTU	Imperial Clinical Trials Unit
IDMC	Independent Data Monitoring Committee
ISRCTN	International standard randomised controlled trial number
MRC	Medical Research Council
MHRA	Medicines and Healthcare products Regulatory Authority
NHS	National Health Service
SAE	Serious Adverse Event
SAR	Serious Adverse Reaction
TMG	Trial Management Group
TSC	Trial Steering Committee

The purpose of this document is to describe the roles and responsibilities of the IDMC for the RIO trial including the timing of meetings, methods of providing information to and from the IDMC, frequency and format of meetings, statistical issues and relationships with other committees.

Content	Charter Details
**1 Introduction**	
**Trial Name**<br>The RIO Trial<br>Sponsor ID: 19IC5249<br>EudraCT: 2019-002129-31	The RIO Trial: A randomised placebo-controlled trial of ART plus dual long-acting HIV-specific broadly neutralising antibodies (bNAbs) vs ART plus placebo in treated Primary HIV Infection on viral control off ART
**Trial objectives (including interventions being investigated)**	To test the hypothesis that for individuals who commenced antiretroviral therapy (ART) in primary HIV infection (PHI), a combination of the two long-acting broadly neutralising antibodies (BNAbs), 3BNC117-LS and 10-1074-LS, will induce a period of virological remission when ART is stopped compared with participants who received ART plus placebo.<br><br>A study summary diagram is included in Appendix I.
Outline of scope of charter	The purpose of this document is to describe the membership, terms of reference, roles, responsibilities, authority, decision-making and relationships of the IDMC for the RIO trial, including the timing of meetings, methods of providing information to and from the IDMC, frequency and format of meetings, statistical issues and relationships with other committees.<br><br>This IDMC charter was developed by the Medical Research Council Clinical Trials Unit and has been adapted by the Imperial Clinical Trials Unit, Imperial College London.
**2 Roles and Responsibilities**	
**A broad statement of the aims of the IDMC**	To safeguard the interests of trial participants, assess the safety and efficacy of the interventions during the trial, and monitor the overall conduct of the clinical trial.
**Terms of reference**	The IDMC should receive and review information on the progress and accruing data of this trial and provide advice on the conduct of the trial to the Trial Steering Committee (TSC) and, indirectly, the Trial Management Group (TMG).<br>The IDMC should inform the Chair of the TSC if, in their view, the results are likely to convince a broad range of clinicians, including those supporting the trial and the general clinical community, that on balance, one trial arm is clearly indicated or contraindicated for all participants or a particular category of participants; and there is a reasonable expectation that this new evidence would materially influence patient management.
**Specific roles of IDMC members**	Interim review of the trial's progress including updated figures on recruitment, data quality, adherence to protocol treatment and follow-up, and main outcomes and safety data. Specifically, these roles may include:

Content	Charter Details
	• Monitor evidence for treatment differences in the main efficacy outcome measures<br>• Monitor evidence for treatment harm (e.g. toxicity, Serious Adverse Events and Serious Adverse Reactions, deaths)<br>• Assess the impact and relevance of external evidence<br>• Decide whether to recommend that the trial continues to recruit participants or whether recruitment should be terminated, either for everyone or for some treatment groups and/or some participant subgroups and/or centres<br>• Decide whether trial follow-up should be stopped<br>• Assess data quality including completeness (and by so doing, encourage collection of high quality data)<br>• Maintain confidentiality of all trial information that is not in the public domain<br>• Monitor recruitment figures and loss to follow-up<br>• Monitor compliance with the protocol by participants and investigators<br>• Consider the ethical implications of their recommendations<br>• Monitor planned sample size assumptions, preferably with regards to<br>(i) Prior assumptions about the control arm outcome and /or<br>(ii) Emerging differences in clinically relevant subgroups, rather than on emerging, unblinded differences between treatment groups, overall<br>• Suggest additional data analyses if necessary<br>• Advise on major protocol modifications proposed by investigators or sponsors (e.g. to inclusion criteria, trial endpoints, or sample size)<br>• Monitor continuing appropriateness of patient information<br>• Monitor compliance with previous IDMC recommendations<br><br>If at any stage an extension to the grant is needed the IDMC may be requested to provide information on the data gathered to date (from this and other studies) and advice on the likelihood that continuation of the trial will allow detection of an important effect. This should be done using methods that do not unblind the trial.
**3 Before or early in the trial**	
**Whether the IDMC will have input into the protocol**	All potential IDMC members should have the opportunity to see the protocol before agreeing to join the committee. Before recruitment begins the trial will have undergone review by the funder / sponsor (e.g. peer review for public sector trials), scrutiny by other trial committees and a research ethics committee (REC). Therefore, if a potential IDMC member has major reservations about the trial (e.g. the protocol or the logistics) they should report these to the trials

Note: This DMC charter was developed using the MRC Clinical Trials Unit template DMC Charter version 2.01, 13-Mar-2006

Content	Charter Details
	unit and may decide not to accept the invitation to join. IDMC members should be independent[1] and constructively critical of the ongoing trial, but also supportive of aims and methods of the trial.
**Whether the IDMC will meet before the start of the trial**	The IDMC will first meet before the trial starts, to discuss the protocol, the trial, future meetings, and to have the opportunity to clarify any aspects with the Chief Investigator (CI) and Trial Management group (TMG). The IDMC should meet within one year of recruitment commencing.
**Whether members of the IDMC will have a contract**	IDMC members will not formally sign a contract. They should formally register their agreement to join the group by confirming (1) that they agree to be on the IDMC and (2) that they agree with the contents of this Charter. Any competing interests should be declared at the same time. Members should complete and return the form in Appendix 2 and retain one copy of the IDMC Charter and the completed form, for their records. Observers attending any part of the meeting should sign a confidentiality agreement on the first occasion they attend all or part of a meeting (Appendix 3).
**4 Composition**	
**Membership and size of the IDMC**	The members of the IDMC for this trial are:<br>(1) *Prof Abdel Babiker, IDMC Chair, Independent statistician*<br>(2) *Prof Jane Anderson, Clinician*<br>(3) *Prof Andrew Lever, Clinician*<br>(4) *Mr Roy Trevelion, Community Representative*<br>The members should be independent of the trial (should not be involved with the trial in any other way or have any competing interest(s) that could impact on the trial). Any competing interests, both real and potential, should be declared. A short competing interest form should be completed and returned by the IDMC members to the trial coordinating centre (Appendix 2).
**The Chair, how they are chosen and the Chair's role.**	The Chair will have previous experience of serving on IDMCs, experience of chairing meetings and will be able to facilitate and summarise discussions. The Chair will be nominated and agreed by the trial team at Imperial College London. The Chair is expected to facilitate and summarise discussions and must attend all IDMC meetings. A vice-Chair will not be appointed.
**The responsibilities of the IDMC Statistician**	The IDMC membership will include a statistician to provide independent statistical expertise, especially with regards to interpretation of accumulating data and guidance through the report. The IDMC statistician will not prepare the IDMC report.
**The responsibilities of the Trial Statistician**	The Trial Statistician will have overall responsibility for the production of the report to the IDMC and will participate in IDMC meetings;

[1] Independence is defined in the table in Appendix IIII

Content	Charter Details
	guiding the IDMC through the report, participating in IDMC discussions and, on some occasions, taking notes.
**The responsibilities of the Trials Unit team**	The Trials Unit team (Trial Manager) will help the trial statistician to produce the report for the IDMC. The Trial Manager and Operations Manager may attend open sessions of the meeting.<br><br>The Trials Unit team (e.g. Trial Manager) will be responsible for running the trial on a day-to-day basis, maintaining trial databases, randomising patients, collating a complete and correct trial dataset; and will help the Trial Statistician to produce the non-confidential sections of the report to the IDMC. The trial team will work with the Joint Research & Compliance Office staff at Imperial College London to deal with research governance and, if appropriate, regulatory matters.
**The responsibilities of the Chief Investigator and other members of the TMG**	The CI should be invited and should be available to attend open sessions of the IDMC meeting. The other TMG members will not usually be expected to attend but can attend open sessions when necessary (see Organisation of IDMC Meetings).
**5 Relationships**	
**Relationships with Chief Investigator, the other trial committees (e.g. Trial Steering Committee), Sponsor and regulatory bodies**	The responsibilities of each trial group are detailed in the protocol. The relationships between these groups are displayed in Appendix 4.
**Clarification on whether the IDMC is advisory (make recommendations) or executive (make decisions)**	The TSC is the oversight body and is delegated this role by the Sponsor. The IDMC is advisory to the TSC. Both the TMG and IDMC make comments, requests and recommendations to the TSC.
**Payments to IDMC members**	Members will be reimbursed for reasonable travel and other expenses incurred in accordance with Imperial College London Expense Policy. No other payments or rewards will be given.
**The need for IDMC members to disclose information about any competing interests**	Competing interests should be disclosed. These are not restricted to financial matters – involvement in other trials or intellectual investment could be relevant. Although members may well be able to act objectively despite such connections, complete disclosure enhances credibility. (See Appendix 2).<br><br>IDMC members should not use interim results to inform trading in pharmaceutical shares, and careful consideration should be given to trading in stock of companies with competing products.
**6 Organisation of meetings**	
**Expected frequency of IDMC meetings**	The IDMC have agreed to meet approximately one month after the first 2 participants have been infused with bNAb/placebo and there-

Content	Charter Details
	after should meet 6 monthly, or more often as appropriate. Meetings should be timed so that reports can be fed into the TSC meetings, and supporting comments added to the annual safety report to the MHRA and REC if applicable. Meetings can be convened at the request of the TMG, Chief Investigators, the Trial Statistician, or any IDMC member. Dates for IDMC meetings should be agreed in advance and only altered with agreement of all members.
**Whether meetings will be face-to-face or by teleconference**	The first meeting should ideally be face-to-face to facilitate full discussion and allow members to get to know each other. It is recommended that all subsequent meetings should be face-to-face if possible, with teleconference as a second option and, ideally, no two consecutive meetings by teleconference.
**How IDMC meetings will be organised, especially regarding open and closed sessions, including who will be present in each session**	The majority of IDMC meetings will be a mixture of open and closed sessions. Only IDMC members and others whom they specifically invite, e.g. the trial statistician, are present in closed sessions. In open sessions, all those attending the closed session may be joined by the CI, other members of the trials unit team and sometimes also representatives of the sponsor, funder or regulator, as relevant. The format of the meetings will be based on the following structure:<br><br>1. Administrative meeting: discussion about any points IDMC would like clarification on in the open session from the investigators (only IDMC members attend, ~5 mins)<br>2. Open session: Introduction and any "open" parts of the report.<br>3. Closed session: IDMC discussion of "closed" parts of the report and, if necessary, the trial statistician will attend only part of these discussions.<br>4. Open session: Discussion with other attendees on any matters arising from the closed session (if necessary).<br>5. Closed session: extra closed session (if necessary).
**7 Trial documentation and procedures to ensure confidentiality and proper communication**	
**Intended content of material to be available in open sessions**	Accumulating information relating to recruitment and data quality (e.g. data return rates, treatment compliance) will be presented. Toxicity details based on pooled data will be presented and total numbers of events for the primary outcome measure and other outcome measures may be presented, at the discretion of the IDMC.<br><br>Tables of all adverse events will be included in the IDMC report, including the following specific AEs based on the approach taken in the US trials of bNAbs:<br><br>Malaise, fatigue, myalgia/arthralgia, feverishness/chills, dizziness or ocular complaints (conjunctival erythema, pain, blurry vision, pruritus), infusion site erythema, induration, pain/tenderness.

Content	Charter Details
**Intended content of material to be available in closed sessions**	In addition to all the material available in the open session, the closed session material will include efficacy and safety data presented by treatment group.
**Whether or not the IDMC will be blinded to the treatment allocation**	The IDMC will not be blinded to treatment allocation.
**The people who will see the accumulating data and interim analysis**	The accumulating data and interim analysis by randomised group will be seen by the IDMC members and the Trial Statistician and his/her delegates.
**Responsibility for identifying and circulating external evidence (e.g. from other trials/ systematic reviews)**	Identification and circulation of external evidence (e.g. from other trials/ systematic reviews) is not the responsibility of the IDMC members. The CI and the ICTU team will collate any such information for presentation in an open session.
**To whom the IDMC will communicate the decisions/ recommendations that are reached**	The IDMC reports its recommendations in writing to the Trial Statistician and, as required, to the Chief Investigator for forwarding to the relevant parties at ICTU. This should be sent to the Trial Statistician and if possible should be sent via the ICTU Trial Manager in time for consideration at a TSC meeting and, where required, for inclusion in the MHRA and REC annual safety reports. A short report/letter to the TMG to say whether the trial should remain unchanged, or whether the matters will be raised with the TSC should be sent via ICTU.<br><br>If the trial is to continue largely unchanged, it is useful for the report from the IDMC to include a summary paragraph suitable for trial promotion purposes i.e. to be circulated to trial sites (See Appendix 5).<br><br>In its communications, the IDMC should be careful not to relay any unnecessary information to the TSC; the TSC membership has independent members but also representatives from the trial team including the Chief Investigator. The IDMC should take care to protect the CI from interim trial data where possible.
**Availability of reports to the IDMC before the meeting**	Where possible, the IDMC should receive the report at least 1 week before any meetings.
**What will happen to the confidential papers after the meeting**	The IDMC members should delete or destroy or store securely the papers after each meeting. After the trial is reported, the IDMC members should destroy all interim reports. A copy of all of the reports will be held by the Trial Statistician separately from the TMF during the course of the study and will be included in the TMF at

Content	Charter Details
	the end of the study. Fresh copies of previous reports may be circulated (by email) with the newest report before each meeting if required.
**8 Decision making**	
**What decisions /recommendations will be open to the IDMC**	Possible recommendations from the IDMC include:-<br>• No action needed, trial continues as planned<br>• Early stopping due, for example, to clear benefit or harm of a treatment, clear lack of benefit or external evidence. (This should generally involve a recommendation to unblind the TSC to this data)<br>• Stopping recruitment within a subgroup (care should be taken if this is not a pre-specified subgroup). (This should generally involve a recommendation to unblind the TSC to this data)<br>• Extending recruitment (based on actual control arm response rates being different to predicted rather than on emerging differences)<br>• Extending follow-up<br>• Proposing or commenting on proposed protocol changes<br>• Commenting on Statistical Analysis Plan
**The role of formal statistical methods, specifically which methods will be used and whether they will be used as guidelines or rules**	Formal statistical methods are more generally used as “stopping” guidelines rather than absolute rules. This is because they generally only consider one dimension of the trial. Reasons should be recorded for disregarding a stopping guideline. The statistical guidelines for the trial are described in outline in the protocol, and in detail in the Statistical Analysis Plan.<br><br>There are no formal statistical stopping guidelines planned for this trial.
**How decisions or recommendations will be reached within the IDMC**	The Chair is to summarise discussions and encourage consensus; it is usually best for the Chair to give their own opinion last.<br><br>Every effort should be made for the IDMC to reach a unanimous decision. If the IDMC cannot achieve this a vote may be taken, although details of the vote should not be routinely included in the report to the TSC as these may inappropriately convey information about the state of the trial data.<br><br>It is important that the implications (e.g. ethical, statistical, practical and financial) for the trial be considered before any recommendation is made.
**When the IDMC is quorate for decision-making**	Every effort should be made to ensure that all members can attend, and the ICTU Team will try to ensure that a date is chosen to enable this. Members who cannot attend in person should be encouraged

Content	Charter Details
	to attend by teleconference. If, at short notice, any IDMC members cannot attend in any capacity then the IDMC may still meet if at least the independent statistician and one clinician, including the Chair (unless otherwise agreed), will be present. If the IDMC is considering recommending major action after such a meeting, the IDMC Chair should communicate with the absent members as soon after the meeting as possible to check they agree; organising a further TC within 3-7 days where necessary. If they do not agree, a further meeting should be arranged with the full IDMC.
**Can IDMC members who cannot attend the meeting input**	If the report is circulated before the meeting, IDMC members who will not be able to attend the meeting may pass comments to the IDMC Chair for consideration during the discussions.
**What happens to members who do not attend meetings**	If a member does not attend a meeting, it should be ensured that the member is available for the next meeting. If a member does not attend the following meeting, they should be asked if they wish to remain part of the IDMC. If a member does not attend a third meeting, they should be replaced using the same election procedure.
**9 Reporting**	
**To whom will the IDMC report their recommendations/decisions, and in what form**	This will be through a letter to the TSC (where applicable) and, on occasion, the Chief Investigator via the Trial Statistician or Trial Manager; usually within 2 weeks of the meeting (see Section 7). A copy of this letter will be lodged with the trial office and filed in the Trial Master File.
**Whether minutes of the meeting be made and, if so, by whom and where they will be kept**	Separate records will be required for open and closed sessions with minutes made by the appropriate attending member of the trial team. This will usually be the Trial Manager for the open session and the Trial Statistician or IDMC member for the closed session. The IDMC Chair should sign off any minutes or notes.
**What will be done if there is disagreement between the IDMC and the body to which it reports**	If the IDMC has serious problems or concerns with a decision made by the TSC, a meeting of both groups should be held. The information to be shown would depend upon the action proposed, and the IDMC's concerns. Depending on the reason for the disagreement, confidential data would often have to be revealed to all those attending such a meeting. The meeting would be Chaired by a senior member of ICTU or an external expert who is not directly involved with the trial.
**10 Release of results**	
**Publication of results**	Depending on the results of the trial and the ease of their interpretation, at the end of the trial there may be a meeting to allow the IDMC to discuss the data with the writing committee to give advice about data interpretation. The main trial results will be published in a correct and timely manner; the TSC should oversee this process.

Content	Charter Details
**The information about the IDMC that will be included in published trial reports**	IDMC members will be named and their affiliations listed in the main report, unless they explicitly request otherwise. A brief summary of the timings and conclusions of IDMC meetings should be included in the body of this paper.
**Whether the IDMC will have the opportunity to approve publications, especially with respect to reporting of any IDMC recommendation regarding termination of a trial**	The IDMC will be given the opportunity to read and comment on publications before submission. This will usually be concurrent with the review by the trial investigators and independent members of the TSC. The commenting period will usually be 2 to 3 weeks.
**Any constraints on IDMC members divulging information about their deliberations after the trial has been published**	The IDMC members may discuss details of their involvement in the study after permission is agreed with the TSC, after the primary results are published

11 Revision History

Version Number	Date Effective	Reason for update
0.1	28 Oct 2019	First draft version of this template
1.0	6 Jan 2020	First approved version

12 Appendix 1: Diagram summarising trial

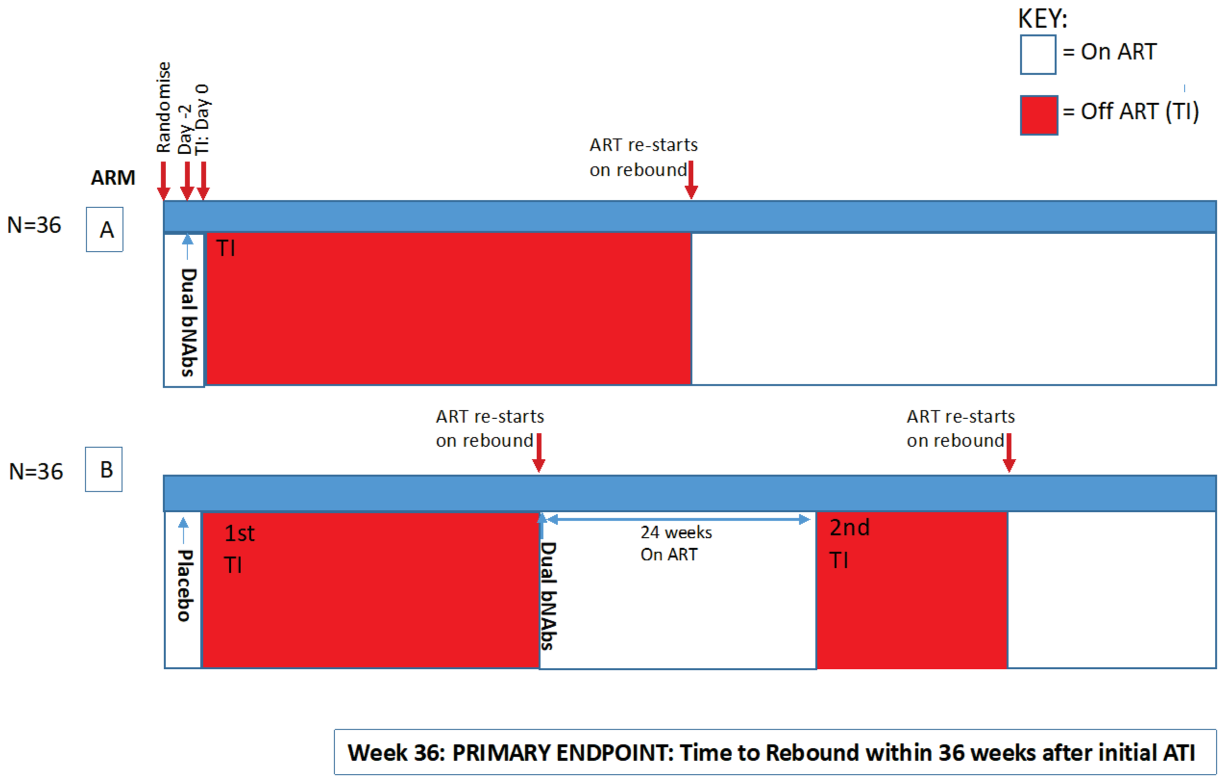

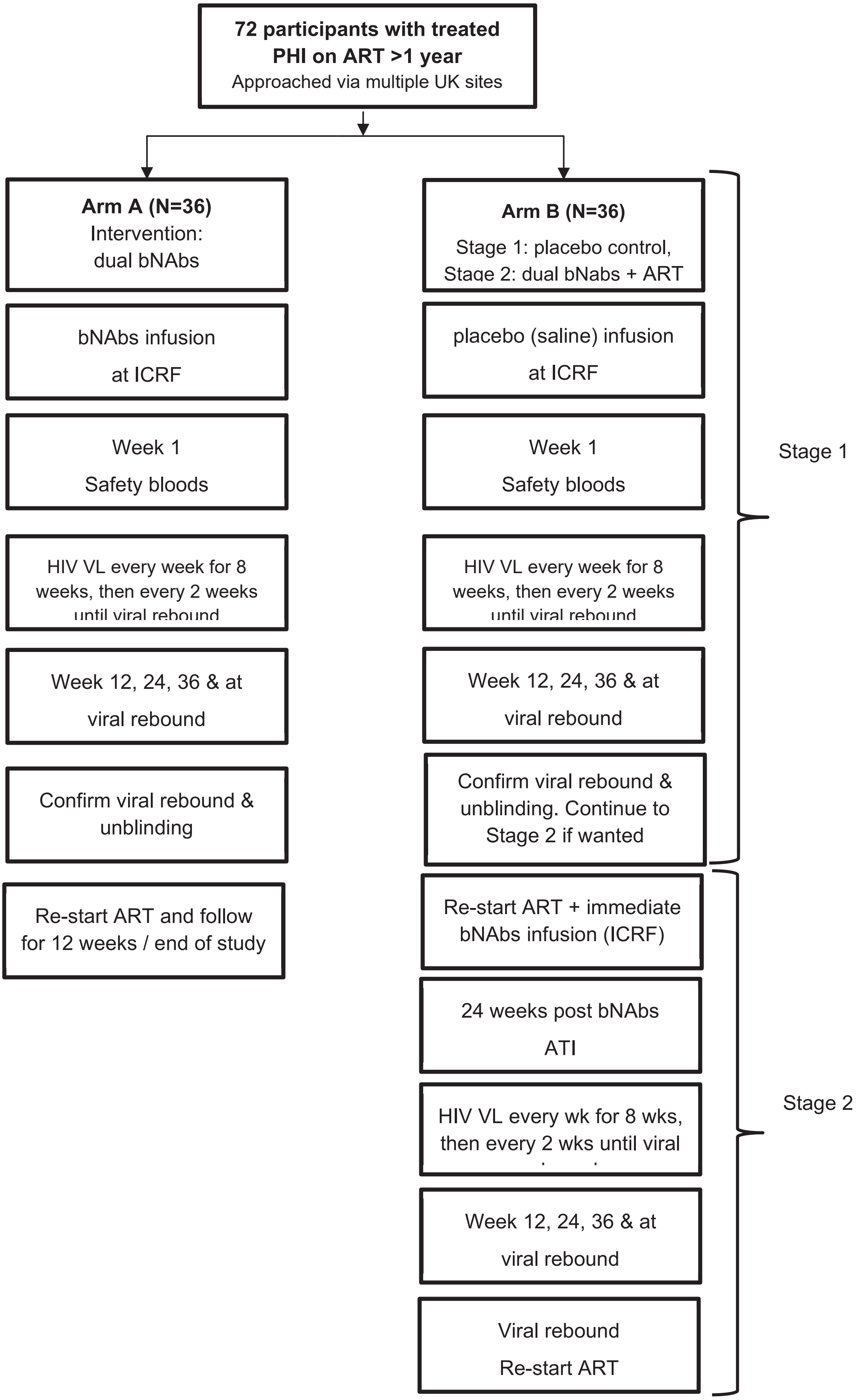
72 participants with treated
PHI on ART >1 year
Approached via multiple UK sites
Arm A (N=36)
Intervention:
dual bNAbs
bNAbs infusion
at ICRF
Week 1
Safety bloods
HIV VL every week for 8
weeks, then every 2 weeks
Week 12, 24, 36 & at
viral rebound
Confirm viral rebound &
unblinding
Re-start ART and follow
for 12 weeks / end of study
Arm B (N=36)
Stage 1: placebo control,
placebo (saline) infusion
at ICRF
Week 1
Safety bloods
HIV VL every week for 8
weeks, then every 2 weeks
Week 12, 24, 36 & at
viral rebound
Confirm viral rebound &
unblinding. Continue to
Stage 2 if wanted
Stage 1
Re-start ART + immediate
bNAbs infusion (ICRF)
24 weeks post bNAbs
ATI
HIV VL every wk for 8 wks,
then every 2 wks until viral
Week 12, 24, 36 & at
viral rebound
Viral rebound
Re-start ART
Stage 2

13 Appendix 2: Agreement and potential competing interests form

Agreement to join the RIO Independent Data Monitoring Committee (IDMC) and disclosure of potential competing interests.

Please complete the following document and return to the Study Team by e-mail (rio_trial@imperial.ac.uk)

(please initial box to agree)

	I have read and understood the IDMC Charter version 1.0, dated 06/01/2020
	I agree to join the IDMC for this trial
	I agree to treat all sensitive trial data and discussions confidentially

The avoidance of any perception that members of a IDMC may be biased in some fashion is important for the credibility of the decisions made by the IDMC and for the integrity of the trial. Possible competing interest should be disclosed via email to the Study Team on (rio_trial@imperial.ac.uk).. In many cases simple disclosure up front should be sufficient. Otherwise, the (potential) IDMC member should remove the conflict or stop participating in the IDMC. Table 1 lists potential competing interests.

	No, I have no competing interests to declare
	Yes, I have competing interests to declare (please detail below)

Please provide details of any competing interests:

____

____

____

Name: ______________________________

Signed: ______________________________ Date: _______________

Table 1: Potential competing interests

- Stock ownership in any commercial companies involved
- Stock transaction in any commercial company involved (if previously holding stock)
- Consulting arrangements with the Sponsor (including CI for other MRC trials)
- Frequent speaking engagements on behalf of the intervention
- Career tied up in a product or technique assessed by trial
- Hands-on participation in the trial
- Involvement in the running of the trial
- Emotional involvement in the trial
- Intellectual conflict e.g. strong prior belief in the trial's experimental arm
- Involvement in regulatory issues relevant to the trial procedures
- Investment (financial or intellectual) or career tied up in competing products
- Involvement in the publication in the form of authorship

14 Appendix 3: Agreement and confidentiality agreement for observers

RIO Independent Data Monitoring Committee: Agreement to attend the IDMC meetings and treat all information confidentially

Please complete the following document and return the Study Team by e-mail (rio_trial@imperial.ac.uk)

(please initial box to agree)

	I have received a copy of the IDMC Charter version 1.0, dated 06/01/2020
	I agree to attend the IDMC meeting on ___/____/______
	I agree to treat as confidential any sensitive trial information gained during this meeting unless explicitly permitted

Name: ___________________________

Signed: __________________________ Date: ______________

15 Appendix 4: Relationship of Trial Committees

The diagram demonstrates the main trial committees, shows how all communications between committees should pass through ICTU (although not always through the same person) and (by use of colour) shows how ICTU is represented on TMG and TSC and how TMG is represented on TSC.

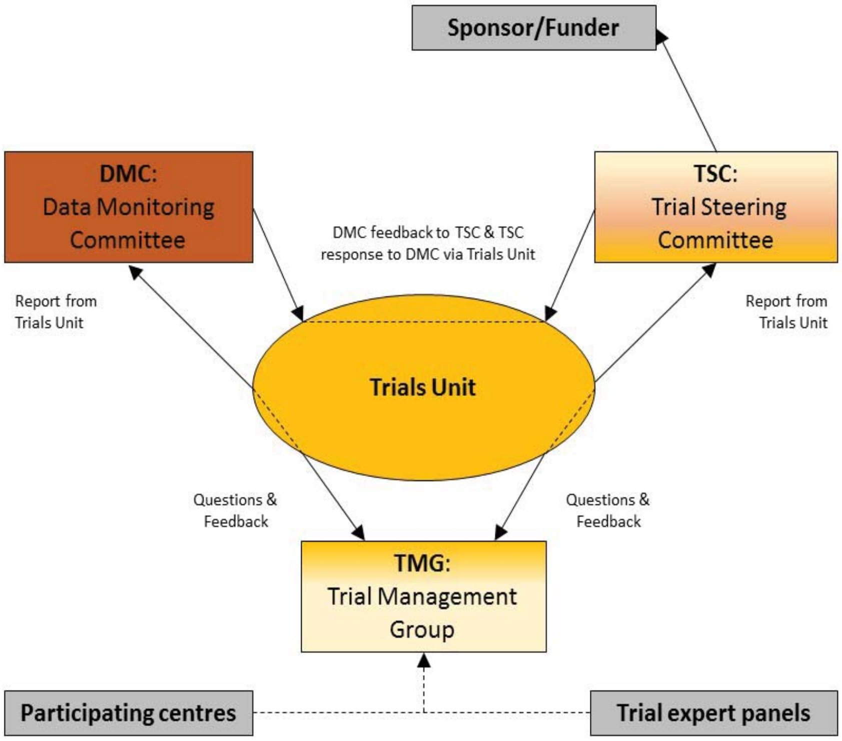

Note: This DMC charter was developed using the MRC Clinical Trials Unit template DMC Charter version 2.01, 13-Mar-2006

16 Appendix 5: Suggested report from IDMC to TSC where no recommendations are being made

[Insert date]

To: Chair of Trial Steering Committee
Via: ICTU Trial Manager

Dear [Chair of Trial Steering Committee]

The Independent Data Safety Monitoring Committee (IDMC) for the RIO trial met on [meeting date] to review its progress and interim accumulating data. [List members] attended the meeting and reviewed the report.

The IDMC would like to congratulate the investigators and trial team on the running of the trial and its recruitment, data quality and follow-up. The trial question remains important and, on the basis of the data reviewed at this stage, we recommend continuation of the trial according to the current version of the protocol [specify protocol version number and date] with no changes.

We shall next review the progress and data [provide approximate timing]

Yours sincerely,

Professor Abdel Babiker
Chair of Independent Data Monitoring Committee

On behalf of the IDMC (all members listed below)

IDMC members:
(1) *Prof Abdel Babiker, IDMC Chair, Independent statistician*
(2) *Prof Jane Anderson, Clinician*
(3) *Prof Andrew Lever, Clinician*
(4) *Mr Roy Trevelion, Community Representative*

17 Appendix 6: Guidelines from US bNAb trials Safety Committee

The Safety Monitoring Committee (SMC) Charter for one of the US trials (Protocol MCA-0965) has been appended to the RIO IDMC charter for reference.
