# Supplementary material for: The RIO trial: rationale, design, and the role of community involvement in a randomised placebo-controlled trial of antiretroviral therapy plus dual long-acting HIV-specific broadly neutralising antibodies (bNAbs) in participants diagnosed with recent HIV infection—study protocol for a two-stage randomised phase II trial
Source: Trials. 2022 Apr 5;23:263. doi: 10.1186/s13063-022-06151-w (PMC8981886; doi:10.1186/s13063-022-06151-w)
Supplement: Supplementary file 4 — Additional file 4. [file 13063_2022_6151_MOESM4_ESM.docx]

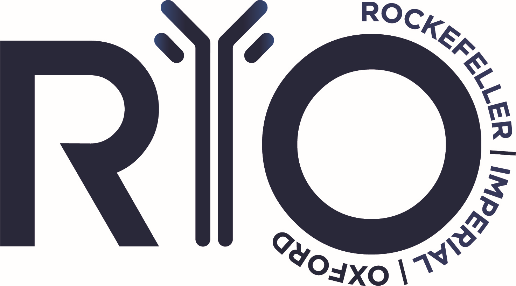
**Chief Investigator: Professor Sarah Fidler**

**Study Protocol Number:** 19IC5249

**IRAS ID:** 266322

**EudraCT:** 2019-002129-31

The RIO Trial: A randomised placebo controlled trial of ART plus dual long-acting HIV-specific broadly neutralising antibodies (bNAbs) vs ART plus placebo in treated Primary HIV Infection on viral control off ART

**Participant Consent Form**

| **Participant Trial ID Number:** |  |
| --- | --- |
| **Site Number/Name:** |  |
| **Name of Principal Investigator:** | **Prof Sarah Fidler** |

**Please initial each box if you agree with the following:**

| 1 | I confirm I have read and understood the Participant Information Sheet, Version ___ dated _ _ /_ _ /_ _ for the above study, have had enough time to review the information and have been able to ask questions which have been answered fully. |  |
| --- | --- | --- |
| 2 | I understand my participation is voluntary and I am free to withdraw consent at any time, without giving any reason, without my medical care or legal rights being affected. |  |
| 3 | I understand my identity will never be disclosed outside of research and any information collected will remain confidential. |  |
| 4 | I understand that sections of any of my medical records and other personal data generated during the study may be examined by representatives of the Sponsor (Imperial College London), by people working on behalf of the Sponsor, or by representatives of regulatory authorities where it is relevant to taking part in this research. I give permission for these individuals to access my records. |  |
| 5 | I consent to the use of my blood samples for the analyses described in the Patient Information Sheet, which includes genetic analysis. I understand that if I withdraw my participation from the study, my samples shall be destroyed if I request this, but the results collected from these samples can still be used. I give permission for my samples to be sent to other organisations, including those outside of the EEA. |  |
| 6 | I give permission for my data to be sent to other organisations, including those outside of the EEA. |  |
| 7 | I give permission for my GP to be informed of my participation in this study  ***(optional)*** |  |
| 8 | I agree to the storage and use of my samples for future ethically approved related research  ***(optional)*** |  |
| 9 | I understand that the information collected about me will be used to support other research in the future, and may be shared anonymously with other researchers  ***(optional)*** |  |
| 10 | I agree to be contacted in the future for research related to this study.  ***(optional)*** |  |
| 11 | I agree to participate in this study. |  |

**Name of Participant Date Signature**

**Name of Person Date Signature**

**taking consent**
